# Transformation of Agro-Industrial By-Products into High-Value Animal-Derived Foods: Bioactive Compounds, Microbiome-Mediated Biotransformation, Metabolomic Traceability and Circular Valorization

**DOI:** 10.3390/molecules31152710

**Published:** 2026-08-04

**Authors:** Lucrezia Forte, Eric N. Ponnampalam, Pasquale De Palo, Edyta Kowalczuk-Vasilev, John Quiñones, Abdelfattah Z. M. Salem, Aristide Maggiolino

**Affiliations:** 1 Department of Veterinary Medicine, University of Bari Aldo Moro, Strada Provinciale per Casamassima Km 3, Valenzano, 70010 Bari, Italy; lucrezia.forte@uniba.it (L.F.); pasquale.depalo@uniba.it (P.D.P.); 2 School of Agriculture, Food and Ecosystems Sciences, The University of Melbourne, Parkville, VIC 3010, Australia; eponnampalam@unimelb.edu.au; 3 Institute of Animal Nutrition and Bromatology, Faculty of Animal Sciences and Bioeconomy, University of Life Sciences in Lublin, 20-950 Lublin, Poland; edyta.kowalczuk@up.edu.pl; 4 Meat Quality Innovation and Technology Centre (CTI-Carne), Universidad de La Frontera, Temuco 4780000, Chile; john.quinones@ufrontera.cl; 5 Faculty of Agricultural and Environmental Sciences, Universidad de La Frontera, Av. Francisco Salazar 01145, Temuco 4780000, Chile; 6 Department of Soil, Plant, and Food, University of Bari Aldo Moro, Via Giovanni Amendola, 165/a, 70126 Bari, Italy; abdelfattah.salem@uniba.it

**Keywords:** circular bioeconomy, feed valorization, foodomics, polyphenols, metabolite transfer, lipid oxidation, gut microbiota, product quality

## Abstract

Agro-industrial by-products are increasingly considered as feed resources for circular animal production, but their value should not be interpreted only as a low-cost replacement of conventional ingredients. This scoping review critically examines how by-products from grape, olive, tomato, citrus, cereal, brewery, oilseed and vegetable processing chains may contribute to the development of high-value animal-derived foods. Particular attention is given to bioactive compounds, polyphenols, carotenoids, tocopherols, fermentable fibres and residual lipids, as well as to microbiome-mediated biotransformation, host metabolic pathways, compound transfer, product-quality modulation, feed safety and circular valorization. Available evidence indicates that selected by-products can influence milk, cheese, meat, eggs and fish products by modifying fatty acid profile, oxidative stability, antioxidant-related traits, pigmentation, volatile compounds, shelf-life and, in some cases, the transfer of specific metabolites to edible products. However, these responses depend on by-product source, processing method, inclusion level, active dose, animal species, basal diet and analytical endpoints. Chemical richness alone is therefore insufficient to support functional claims. Stronger evidence requires studies that connect matrix characterization, processing stability, microbial and host-mediated transformation, biological intermediates and final product quality within the same experimental design. Precision circular feeding should therefore combine local availability, safety, active-dose definition, metabolomic and lipidomic traceability, and product-level validation to support reproducible improvements in high-value animal-derived foods.

## 1. Introduction

Agro-industrial by-products are increasingly used as feed resources to support circular animal production systems and ecosystem protection, but their value should not be reduced to the replacement of conventional feed ingredients alone. Fruit pomaces, olive mill residues, cereal brans, brewer’s spent grain, oilseed cakes, citrus pulp, tomato pomace and other secondary streams can recover nutrients that would otherwise remain underused, while also reducing disposal pressure and partially easing feed–food competition [1,2,3]. Beyond their nutritional contribution, these matrices may contain polyphenols, including phenolic acids, flavonoids, tannins and stilbenes, as well as carotenoids, terpenes, tocopherols, unsaturated lipids, fermentable fibres and residual proteins able to interact with digestion, microbial metabolism, host physiology and product quality [3,4,5]. They should therefore be interpreted as complex molecular matrices, not only as low-cost or alternative feed ingredients, but also as potential modulators of animal metabolism and the quality of milk, meat, eggs, fish and aquaculture products.

However, the inclusion of a by-product in an animal diet does not automatically generate a functional animal-derived food. In this review, the term functional animal-derived food is used in an operational scientific sense, rather than as a regulatory health claim. It refers to milk, cheese, meat, eggs, fish or derived products in which feeding strategies based on agro-industrial by-products produce measurable nutritional, molecular, oxidative, technological, sensory or shelf-life-related improvements, or lead to the presence of specific dietary, microbial or host-derived metabolites. These dimensions should not be considered equivalent: direct enrichment with a defined compound, metabolite-mediated transfer and indirect modulation of product quality represent different levels of evidence. Many feeding studies evaluate by-products mainly through intake, digestibility, growth performance, feed efficiency or milk yield, whereas fewer studies connect the chemical profile of the by-product with measurable changes in milk, cheese, meat, eggs or fish products [6,7,8]. Productive outcomes remain essential to define the feasibility of a feeding strategy, but they are not sufficient to demonstrate that the final product has acquired additional nutritional, antioxidant, sensory, technological or health-related value [9,10]. A further limitation of the available literature is that relatively few studies simultaneously evaluate by-product characterization, processing stability, microbiome-mediated transformation, host metabolism and final product traits within the same experimental design.

This limitation is central because the biological activity of a by-product also depends on what happens after processing, ingestion and digestion. After ingestion, polyphenols may be released from the plant cell wall, degraded by ruminal or intestinal microorganisms, transformed into smaller phenolic acids, conjugated by the host, or bound to proteins and fibres before they can influence edible tissues or secretions [11]. Carotenoids and other lipophilic compounds may be affected by matrix structure, dietary lipid supply, oxidative stability and species-specific absorption mechanisms before they are deposited in milk fat, egg yolk or animal tissues [3,9]. Thus, the functional potential of a by-product depends less on its nominal richness in bioactive compounds and more on bioaccessibility, microbial conversion, host utilization and reproducible effects in the final animal-derived food [12].

The microbiome is a central intermediary in this process. In ruminants, ruminal microorganisms degrade and transform plant compounds, ferment structural carbohydrates, modify nitrogen metabolism and drive the biohydrogenation of unsaturated fatty acids, thereby influencing methane formation, nitrogen use, milk fatty acid profile and meat lipid composition [13,14,15]. In monogastric species, intestinal microbiota can transform fibres, oligosaccharides and polyphenols into short-chain fatty acids and low-molecular-weight metabolites with potential systemic effects [11]. For this reason, the microbiome should be considered a functional filter between feed composition and product quality, rather than only a response variable.

Current evidence suggests that selected agro-industrial by-products can improve specific traits of animal-derived foods, including fatty acid profile, oxidative stability, antioxidant-related traits, color stability, volatile compound formation, shelf-life and, in some cases, the presence of specific metabolites in the final product [6,9,16]. However, these effects are context-dependent. They vary according to by-product source, processing method, inclusion level, active dose, animal species, basal diet and analytical endpoints [8]. Processing adds further complexity, because drying, milling, ensiling, fermentation, enzymatic treatment and green extraction can preserve, release, concentrate or degrade bioactive fractions while also modifying storage stability, digestibility, cost, safety and environmental impact [3,17,18].

**Figure 1:**
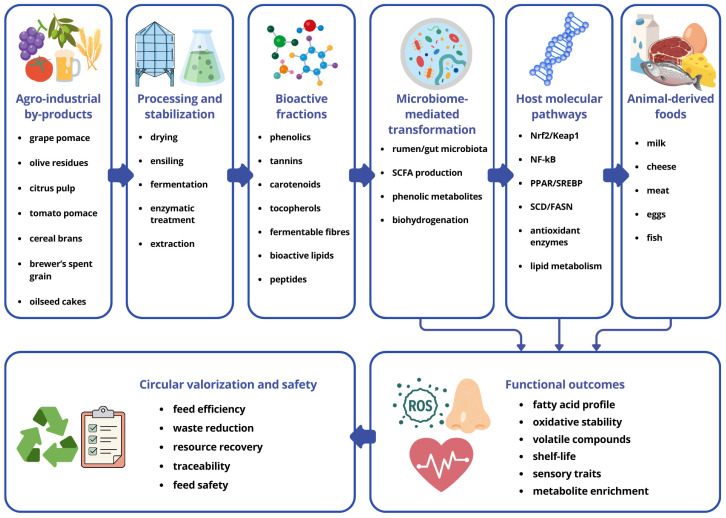
Conceptual framework linking agro-industrial by-products with functional animal-derived foods through processing, microbiome-mediated biotransformation, host molecular pathways and circular valorization.

The conceptual framework of this review follows the sequence from by-product composition to processing, microbiome-mediated biotransformation, host metabolism, compound transfer or indirect modulation, animal-derived food quality and circular valorization (Figure 1). The main classes of by-products, their dominant bioactive fractions, expected molecular pathways and critical limitations are summarized in Table 1. This review therefore aims to critically examine how agro-industrial by-products may contribute to the development of functional and high-value animal-derived foods through bioactive compounds, microbiome-mediated biotransformation, host metabolic pathways, metabolomic traceability and circular valorization. Particular attention is given to the distinction between direct transfer of dietary compounds, transfer of microbial or host-derived metabolites, and indirect modulation of lipid metabolism, oxidative balance, volatile profile, technological traits and shelf-life.

## 2. Materials and Methods

### 2.1. Review Design and Reporting Framework

This review was conducted as a scoping review with critical narrative synthesis. This design was selected because the literature on agro-industrial by-products in animal feeding is highly heterogeneous in terms of animal species, by-product matrices, processing methods, inclusion levels, feeding duration, analytical endpoints, and product-quality outcomes. The aim was not to estimate pooled effects, but to map the available evidence, identify recurring molecular and biological mechanisms, and define knowledge gaps limiting the translation of by-product feeding into functional animal-derived foods. The review followed the PRISMA extension for Scoping Reviews, PRISMA-ScR, with additional reference to PRISMA 2020 for record identification, screening and inclusion reporting, and to Joanna Briggs Institute guidance for the definition of the review question, eligibility criteria, data charting and evidence synthesis [35,36,37].

### 2.2. Review Questions

The review questions were structured according to the Population–Concept–Context framework. The population included food-producing animals, including ruminants, pigs, poultry, rabbits, fish and other aquaculture species, when the final product was intended for human consumption. The concept was the use of agro-industrial by-products, food-chain residues or processed fractions as feed ingredients or bioactive sources able to influence animal metabolism and product quality. The context was the production of milk, cheese, meat, eggs, fish and derived products with measurable nutritional, molecular, oxidative, sensory, technological or shelf-life-related outcomes. The review was guided by six questions:

- which agro-industrial by-products have been investigated as feed resources or bioactive matrices?
- which molecular fractions are most frequently involved?
- which ruminal, intestinal, microbial or host-mediated pathways have been proposed?
- which effects have been reported on animal-derived food quality?
- which methodological gaps limit the development of credible functional-food interpretations?
- which safety, regulatory, logistical and circularity-related factors influence the practical translation of by-product feeding strategies into high-value animal-derived foods?

### 2.3. Information Sources and Search Strategy

A structured literature search was performed in Scopus, PubMed/MEDLINE and Web of Science Core Collection. The search covered papers published from 1 January 2011 to 15 June 2026. Older articles were considered only when they provided essential methodological or conceptual information that was not replaced by more recent literature. The final search was updated on 15 June 2026. Search terms were combined into five blocks: agro-industrial by-products, animal feeding, bioactive compounds, microbial or host-mediated biotransformation, and animal-derived food quality. The by-product block included terms such as “agro-industrial by-product*”, “agri-food by-product*”, “food processing residue*”, “grape pomace”, “olive pomace”, “olive mill wastewater”, “tomato pomace”, “citrus pulp”, “cereal bran”, “brewer’s spent grain”, “oilseed cake” and “vegetable residue*”. The animal-feeding block included “animal feed”, “livestock”, “ruminant*”, “dairy cow*”, “sheep”, “goat*”, “pig*”, “poultry”, “laying hen*”, “rabbit*”, “fish” and “aquaculture”. The molecular and mechanistic blocks included terms related to polyphenols, tannins, carotenoids, tocopherols, fatty acids, peptides, metabolomics, lipidomics, microbiome, fermentation, biohydrogenation, short-chain fatty acids, urolithins, equol and phenolic metabolites. The product-quality block included “milk quality”, “cheese”, “meat quality”, “egg quality”, “fish quality”, “oxidative stability”, “fatty acid profile”, “color stability”, “volatile compounds”, “sensory quality”, “shelf-life” and “functional food*”. The full database-specific search strings, including field codes and filters, are reported in Supplementary Table S1.

Reference lists of eligible articles and relevant reviews were manually screened, and forward citation tracking was used for selected papers dealing with microbiome-mediated transformation, metabolomic tracing, lipid biohydrogenation, product-quality modulation and circular valorization. Safety-related information was specifically checked during full-text assessment and data charting, using terms related to feed hygiene, mycotoxins, pesticide residues, heavy metals, microbial contamination, spoilage risk and solvent residues. These terms were used to support the safety-oriented interpretation of eligible studies and contextual sources, without changing the main PRISMA record count.

### 2.4. Eligibility Criteria and Study Selection

**Figure 2:**
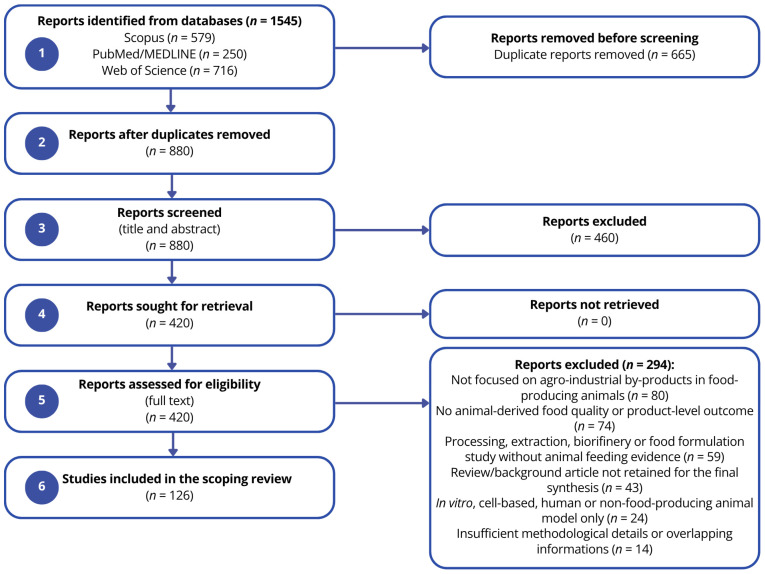
PRISMA flow diagram showing identification, screening, eligibility assessment and inclusion of studies in the scoping review.

Peer-reviewed original articles were included when they evaluated agro-industrial by-products, food-chain residues or processed fractions in the diet of food-producing animals and reported at least one outcome related to animal-derived food quality, bioactive compounds, microbiome, metabolism, oxidative stability, fatty acid profile, sensory traits, technological properties, safety or sustainability. Whole feed matrices, stabilized ingredients, fermented products, enzymatically treated materials, ensiled fractions and concentrated bioactive sources derived from agro-industrial residues were considered eligible. In vivo feeding trials were considered the strongest source of evidence when they linked by-product inclusion with animal response and measurable traits of milk, cheese, meat, eggs, fish or derived products. In vitro rumen fermentation, gut fermentation, enzymatic digestion, simulated digestion, extraction and biotransformation studies were also included when they provided mechanistic information useful for interpreting in vivo or product-quality outcomes. Reviews and meta-analyses were used to frame the topic, identify gaps and support mechanistic interpretation. Studies were excluded when they focused only on agronomic production, composting, bioenergy, packaging, waste disposal or non-food uses of residues without a clear connection to animal feeding or animal-derived foods. Conference abstracts, theses, patents, book chapters, non-peer-reviewed reports, ResearchGate-only records and non-English papers were excluded from the main evidence synthesis. Institutional or regulatory documents were used only when feed safety, legal classification or regulatory interpretation could not be adequately supported by peer-reviewed literature. Eligibility criteria and data-charting categories were defined before full screening. Title and abstract screening was performed by the main reviewers, and uncertain records were discussed until consensus was reached. Full-text eligibility was assessed using the same criteria, with particular attention to the connection between by-product use, animal feeding, mechanistic evidence and animal-derived food outcomes. No formal protocol was registered. However, the screening process, search strings, eligibility criteria and reasons for exclusion were documented to improve transparency and reproducibility. All records retrieved from Scopus, PubMed/MEDLINE and Web of Science Core Collection were exported with the available bibliographic metadata and merged into a single reference file. The database search identified 1545 records: 579 from Scopus, 250 from PubMed/MEDLINE and 716 from Web of Science Core Collection. After removal of 665 duplicate records, 880 records were screened by title and abstract. Of these, 460 records were excluded because they were outside the scope of the review. Full texts were sought for 420 reports, all of which were retrieved and assessed for eligibility. After full-text assessment, 294 reports were excluded, mainly because they were not focused on dietary use of agro-industrial by-products in food-producing animals, did not report animal-derived food quality or product-level outcomes, dealt only with processing, extraction, biorefinery or food formulation without animal feeding evidence, or were reviews/background articles not retained for the final synthesis. Finally, 126 sources were included in the review. The study-selection process is summarized in the PRISMA flow diagram shown in Figure 2.

### 2.5. Data Charting and Synthesis

Data were charted using a structured extraction form. The standardized data extraction template used to organize bibliographic, nutritional, mechanistic, product-quality, safety and circularity information is reported in Supplementary Table S4. Extracted information included author, year, country, animal species, production system, by-product type, botanical or industrial origin, stabilization method, processing strategy, inclusion level, feeding duration and basal diet characteristics. When available, information was also extracted on proximate composition, fibre fractions, lipid profile, phenolic profile, carotenoid content, antioxidant capacity, anti-nutritional factors, contaminants and analytical methods. Microbiome-related information included ruminal or intestinal microbial composition, fermentation variables, biohydrogenation indicators, short-chain fatty acids, microbial metabolites and omics-based endpoints. Host-response information included productive performance, metabolic indicators, oxidative-stress markers, inflammatory markers, lipid metabolism, immune response and transcriptomic, metabolomic, lipidomic or proteomic data. Product-quality information included milk composition, cheese traits, meat quality, egg quality, fish fillet quality, fatty acid profile, lipid and protein oxidation, antioxidant capacity, color stability, volatile compounds, sensory traits, shelf-life and technological properties. Safety and circularity information included feed hygiene, mycotoxins, pesticide residues, heavy metals, spoilage risk, processing intensity, substitution of conventional feedstuffs, local availability, scalability, life-cycle indicators and regulatory considerations. No quantitative meta-analysis was performed due to the heterogeneity of species, by-products, inclusion levels, processing methods, trial duration, analytical methods and outcome definitions. Evidence was synthesized narratively along the feed–microbiome–host–food continuum, distinguishing direct transfer of native dietary compounds, transfer of microbial or host-derived metabolites, and indirect modulation of product quality through changes in lipid metabolism, oxidative balance, inflammatory status, ruminal biohydrogenation or gut fermentation.

### 2.6. Strength of Interpretation

The strength of evidence was interpreted using a semi-quantitative framework developed for this scoping review and summarized in Table S2A–C. Five domains were considered: by-product characterization and active-dose definition; feeding design and biological relevance; final animal-derived product analysis; microbial, metabolic or host-mediated mechanistic evidence; and safety or circularity reporting. Each domain was scored from 0 to 2, where 0 indicated absent or insufficient information, 1 indicated partial information, and 2 indicated adequate information. The total score therefore ranged from 0 to 10. Evidence was considered strong when the total score was 8–10, moderate when it was 6–7, weak when it was 3–5, and insufficient for functional-food interpretation when it was 0–2. This scoring system was not intended to perform a quantitative meta-analysis or to rank studies in a statistical sense. Rather, it was used to harmonize the interpretation of heterogeneous evidence and to distinguish studies supporting direct enrichment, metabolite-mediated transfer, or indirect modulation of product quality from studies reporting only chemical composition, extraction yield, in vitro antioxidant capacity, or general performance data.

## 3. Chemical and Molecular Diversity of Agro-Industrial By-Products

### 3.1. By-Products Are Not a Homogeneous Class of Feed Ingredients

The term “by-product” describes the origin of a material but says little about its nutritional role or biological actions in the animal upon consumption. Grape pomace, olive pomace, olive leaves, citrus pulp, tomato pomace, cereal brans, brewer’s spent grain, oilseed cakes and vegetable residues differ widely in dry matter, protein, fibre, lipid fraction, phenolic profile, carotenoid content, fermentability and storage stability. Their composition also changes with cultivar, harvest maturity, industrial process, recovered fraction, drying method, particle size, storage time and moisture level. As a result, two materials belonging to the same by-product category may behave as different feed matrices once included in a diet [1,2,3]. This variability has direct implications to produce functional animal-derived foods. Some by-products serve mainly as sources of fibre, residual energy or protein, whereas others also supply bioactive compounds that may influence microbial metabolism, oxidative balance, lipid metabolism or product quality. Phenolic acids, tannins, flavonoids, carotenoids, tocopherols, terpenes, fermentable polysaccharides, unsaturated lipids and residual peptides may all contribute to biological responses, but their relevance depends on concentration, accessibility, stability and dose [3,4,9]. Proximate composition remains essential for diet formulation, but it cannot alone explain why a by-product modifies the fatty acid profile, oxidative stability, volatile compounds, color, sensory traits or technological properties of milk, cheese, meat, eggs or fish. When functional effects are expected, compositional data should be complemented by molecular descriptors such as individual phenolic compounds, tannin fractions, carotenoids, tocopherols, lipid quality, fibre structure, fermentability, antioxidant capacity, anti-nutritional factors and contaminants [1,9,11]. Without this information, it is difficult to separate the effect of a specific bioactive fraction from other influencing factors such as energy supply, fibre replacement, lipid addition or matrix interactions. For this reason, studies should report, at minimum, the botanical or industrial origin of the by-product, recovered fraction, processing and stabilization method, storage conditions, dry matter content, inclusion level on a dry-matter basis, main nutritional fractions and the concentration of the bioactive compounds or compound classes used to support functional interpretation. When possible, these data should be reported for the final feed ingredient as administered to animals, not only for the raw residue. Processing can further change the meaning of the same residue. Dried grape pomace, for example, may differ in nutritional value, phenolic profile and in vitro digestion behavior according to micronization and air-classification protocols, showing that technological fractionation can reshape a winery residue into matrices with different feeding potential [38]. The main by-products considered in this review, along with their dominant bioactive fractions, expected molecular relevance and main limitations, are summarized in Table 1.

### 3.2. Polyphenols and Tannins: Relevant, but Difficult to Interpret

Polyphenols are among the most frequently discussed bioactive fractions in agro-industrial by-products used to enhance feed value and product quality. They occur in grape pomace, grape seeds, olive leaves, olive pomace, olive mill wastewater, pomegranate residues, citrus peels and several vegetable by-products. This broad group includes phenolic acids, flavonoids, tannins, stilbenes, lignans and secoiridoid derivatives, which differ in polarity, solubility, protein-binding capacity and susceptibility to microbial metabolism [2,9,11]. The biological interest in phenolic-rich by-products is often linked to antioxidant, anti-inflammatory and antimicrobial properties, but in food-producing animals the response is rarely explained by direct antioxidant activity alone. In ruminants, phenolic compounds and tannins may interact with rumen microorganisms, bind dietary proteins, alter nitrogen metabolism, influence fibre degradation and modify lipid biohydrogenation. In monogastrics, intestinal microbiota can degrade phenolic compounds into smaller metabolites with different absorption rates and biological activities. The response therefore depends on chemical structure, dose, matrix accessibility, microbial metabolism and basal diet, rather than on total phenolic content alone [11,13]. Grape pomace illustrates this complexity well. It may contain condensed tannins, anthocyanins, flavanols, phenolic acids, stilbenes and residual seed lipids, but its composition varies with grape variety, winemaking process, seed-to-skin ratio, drying conditions and storage. Recent literature describes grape pomace as a promising feed enrichment tool for improving the quality of foods of animal origin, although responses depend on inclusion level, animal species, polyphenol supply, fibre content and palatability [19]. Technological fractionation adds another layer of variability, since micronization and air classification can modify both phenolic traits and the digestion behavior of dried grape pomace [38]. Tannins require particular caution because their effects are dose- and matrix-dependent. At moderate levels, they may reduce excessive ruminal protein degradation, influence microbial lipid biohydrogenation and contribute to a more favorable fatty acid profile in milk or meat. At higher levels, or when highly reactive tannin fractions are used, they may depress feed intake, fibre digestion or nutrient availability. Tannin-rich by-products should therefore not be presented generically as beneficial natural antioxidants; the same class of compounds may produce positive, neutral or negative responses depending on dose, basal diet, animal species and tannin structure [13,14,39]. Olive-derived residues raise a similar issue. Olive pomace, olive cake, olive leaves and olive mill wastewater may contain hydroxytyrosol, tyrosol, oleuropein derivatives, verbascoside and other secoiridoid-related compounds, but their relative abundance depends on cultivar, oil extraction process and recovered fraction. These residues have been associated with changes in rumen function, oxidative status and milk or meat fatty acid profile, especially in ruminants, although the strength of evidence varies with the degree of matrix, level of inclusion in the ration and product characterization [20]. Stronger evidence is provided when compounds or metabolites are measured in the final food. In dairy sheep, supplementation with an olive mill wastewater polyphenolic extract was associated with hydroxytyrosol, tyrosol and related metabolites in cheese [21]. Similar compounds have also been detected in Italian cheeses, confirming that olive-derived polyphenols can be traced in dairy matrices when sensitive analytical methods are carried out [22]. Citrus by-products have a different phenolic profile, with pectin, soluble carbohydrates, flavanone glycosides such as hesperidin and naringin, and volatile terpenes such as limonene. In dairy cows, pelleted citrus pulp included at 9% or 18% of dietary dry matter increased total polyphenols, flavonoids, and ferric reducing antioxidant power in milk, providing a clear example of a by-product affecting antioxidant-related traits in the final product [23]. However, this type of response does not necessarily identify which compounds or metabolites were transferred or responsible for the antioxidant effect. Meta-analytic evidence in dairy sheep and goats further supports the potential of phenolic-rich by-products to reduce saturated fatty acids and increase nutritionally relevant unsaturated fatty acids in milk, including vaccenic, rumenic and linolenic acids, but the magnitude of the response varies with species, source of polyphenols, inclusion level, polyphenol dose and basal diet [16].

### 3.3. Carotenoids, Tocopherols and Other Lipophilic Bioactives

Carotenoids and other lipophilic molecules represent a second major group of bioactive compounds in agro-industrial by-products. They are particularly relevant in tomato pomace, carrot residues, citrus by-products, leafy vegetable residues and oil-rich seed fractions. Tomato pomace is a useful example because it contains lycopene, β-carotene, fibre, seed oil, tocopherols and some phenolic compounds, making it a mixed matrix rather than a simple carotenoid source [24,40]. The interpretation of carotenoid-rich residues should consider matrix release and lipid-mediated absorption. Lycopene and β-carotene are lipophilic molecules whose bioaccessibility depends on disruption of the plant matrix, particle size, drying temperature, oxidative conditions, dietary fat level, and micellar incorporation. High carotenoid concentration in the feed may have limited biological relevance if these compounds remain trapped in the matrix or are degraded during processing and storage. Conversely, moderate concentrations may become more relevant when processing improves release, and the diet supports absorption [24,26]. Eggs provide one of the clearest models for evaluating carotenoid transfer from feed to animal-derived foods. In laying hens, tomato powder supplementation increased carotenoids and vitamin A in egg yolk and reduced yolk lipid peroxidation, showing a direct connection between a carotenoid-rich feed matrix and an enriched animal product [25]. A meta-analysis on tomato waste supplementation in laying hens reported positive effects on performance and egg quality, supporting the practical value of tomato-derived residues in poultry nutrition [41]. More broadly, natural carotenoid supplementation has been associated with improved egg quality, pigmentation and selected immune-related outcomes, although effects depend on source, dose, basal diet and experimental conditions [42]. Recent reviews also indicate that eggs, milk and meat can act as carriers for carotenoids, but enrichment level differs widely among species and tissues [26]. Tocopherols, phytosterols and minor lipid compounds deserve attention in oilseed cakes, grape seeds, tomato seeds and other residues containing residual lipid fractions. These compounds may support antioxidant protection and lipid quality, but they are not always measured in feeding trials. Lipid-rich by-products can improve the fatty acid profile of milk, meat or eggs, yet increased unsaturation may also increase susceptibility to lipid oxidation when antioxidant protection is inadequate [43]. For this reason, fatty acid profile and oxidative stability should be evaluated together rather than treating improved unsaturation as an isolated benefit [6,9,16,44,45]. Terpenes and terpenoids occur in several residues, especially citrus peels and olive-derived matrices. Citrus residues contain volatile monoterpenes, mainly limonene and related compounds, whereas olive residues may contain secoiridoid derivatives and triterpenic acids. These molecules may contribute to antimicrobial or antioxidant effects, but they may also affect palatability and sensory traits of animal-derived foods when used at high inclusion levels. This is particularly relevant for aromatic residues, where biologically interesting molecules may also create flavor-transfer problems or reduce intake if the dose is not controlled [3,20,46].

### 3.4. Fibre-Bound Phenolics, Oligosaccharides, Proteins and Bioactive Lipids

The functional value of by-products is not limited to molecules usually classified as antioxidants. Fibres, oligosaccharides, resistant starch fractions, residual proteins, peptides and bioactive lipids may act through microbial fermentation, gut health, nutrient partitioning and lipid metabolism. Citrus pulp, apple pomace and several vegetable residues are rich in pectins and soluble carbohydrates, whereas cereal brans and brewer’s spent grain provide structural carbohydrates, residual proteins and phenolic acids bound to cell-wall polysaccharides [4,7,31]. These components may not transfer directly to the final food, but they can still influence the biological pathways that shape product quality. Cereal brans are rich in fibre, minerals and phenolic acids, especially ferulic acid, which is largely located in the outer layers of the grain. In wheat bran, phenolic acids are often bound to arabinoxylans and other cell-wall structures, limiting immediate bioaccessibility but creating a substrate for microbial or enzymatic release [28,30]. Brewer’s spent grain follows a similar logic. It is a major brewing by-product, rich in fibre and protein, with arabinoxylans, cellulose, lignin and residual polyphenols as relevant fractions. Its use as feed is well established, but its potential as a source of bioactive compounds depends on processing because many phenolic acids remain bound to the lignocellulosic matrix [31,32]. Arabinoxylans provide a useful connection between fibre chemistry, microbiome activity and host response. Cereal-derived arabinoxylans can be fermented by gut microbiota, producing short-chain fatty acids and influencing microbial community composition, intestinal barrier function and host metabolism. Ferulic acid bound to arabinoxylans may also contribute to prebiotic-like responses, indicating that fibre-bound polyphenols may act through combined microbial and molecular mechanisms rather than as free antioxidants alone [29]. This is particularly relevant for monogastric species, where hindgut fermentation can convert fibre-rich residues into microbial metabolites with systemic effects. In ruminants, fibre-rich by-products may influence product quality through ruminal fermentation rather than through direct transfer of recognizable compounds. Carbohydrate structure can affect volatile fatty acid production, microbial protein synthesis, ruminal pH, methane production and lipid biohydrogenation, with potential consequences for milk fat synthesis and meat fatty acid profile. However, the connection between fibre structure, rumen microbiota and final product quality is still less clearly demonstrated than that between dietary lipids and enrichment of fatty acids in ruminant products. Functional claims for fibre-rich residues should therefore be cautious unless ruminal fermentation, microbial data and product-quality endpoints are evaluated together [4,13]. Residual proteins and peptides are relevant in oilseed cakes, brewer’s spent grain, legume by-products and fruit seed fractions. Their feeding value depends on protein digestibility, heat damage, anti-nutritional factors and their capacity to replace conventional protein sources. In monogastrics and aquaculture, bulky fibre-rich residues may require processing to improve digestibility and reduce anti-nutritional constraints. In ruminants, these matrices can often be included more easily, but their effects on nitrogen metabolism, microbial protein synthesis and product quality still require careful evaluation, as dietary protein can be degraded and partly converted into microbial protein during ruminal fermentation [3,7,31]. Bioactive lipids create an additional link between feed composition and product quality. Oilseed cakes, grape seeds, tomato seeds and some fruit kernels can provide unsaturated fatty acids, tocopherols, phytosterols and other minor lipid compounds. These fractions may improve the fatty acid profile of milk, meat or eggs, but they may also increase susceptibility to lipid oxidation if antioxidant protection is inadequate during post-harvesting or storage. This metabolic trade-off necessitates an integrated approach to prevent post-harvest quality deterioration [6,9,16,47].

### 3.5. From Chemical Richness to Demonstrated Functionality

Chemical richness is only the starting point. A residue rich in polyphenols, carotenoids, fibres, antioxidant compounds or unsaturated lipids can support the development of functional animal-derived foods only if the relevant fraction survives processing, becomes bioaccessible, is transformed and absorbed in biologically meaningful forms, and is associated with measurable changes in milk, cheese, meat, eggs, fish or derived products [9,11]. Only a limited number of studies follow this chain with enough detail. Citrus pulp supplementation in dairy cows increased total polyphenols, flavonoids and ferric reducing antioxidant power in milk, linking a specific by-product with antioxidant-related traits in the final product [23]. Olive mill wastewater polyphenolic supplementation in dairy sheep was associated with hydroxytyrosol, tyrosol and related metabolites in cheese, providing stronger evidence of molecular transfer from a by-product-derived fraction to a dairy product [21,22]. Tomato powder supplementation in laying hens increased yolk carotenoids and vitamin A while reducing yolk lipid peroxidation, showing a relatively direct connection between carotenoid-rich feed and egg enrichment [25]. Representative studies in which agro-industrial by-products were linked with measurable product-level or functional traits are summarized in Table 2. These examples show that the most promising by-products are not necessarily those with the highest concentration of a single bioactive compound. More relevant are matrices whose molecular profile is characterized, stable after processing, bioaccessible, safe, and linked to reproducible effects in animal-derived foods. A by-product may be circular and nutritionally valuable without necessarily supporting the production of a functional food. Functional effects should therefore be claimed only when the pathway from matrix composition to animal response and final product quality is sufficiently supported [7,9].

## 4. Processing and Biotechnological Strategies to Preserve, Release and Upgrade By-Product Bioactives

### 4.1. Processing Is Part of Functional Value, Not Only a Practical Step

The functional potential of agro-industrial by-products is shaped by the way they are stabilized, processed and stored before inclusion in animal diets. Residues may contain polyphenols, carotenoids, lipids, fibres or peptides after industrial processing, but these fractions can be oxidized, degraded, polymerized, fermented by uncontrolled microorganisms or made poorly available during storage and handling. Processing therefore contributes directly to the biological value, safety and consistency of the final feed matrix [3,57]. This is especially relevant for high-moisture residues such as fruit pomaces, tomato pomace, citrus pulp, olive residues, vegetable wastes and mixed agro-industrial streams. These materials are often seasonal, rich in water and susceptible to rapid spoilage. Drying, ensiling, fermentation, milling and extraction can extend their usable life and improve diet formulation, but each strategy modifies the original matrix differently. The processed ingredient should therefore be evaluated as a new feed material, not as a chemically identical version of the fresh residue [3,7]. Three processing levels can be distinguished. The first includes simple stabilization methods, such as drying, milling, pelleting and ensiling, where the whole matrix is largely retained. The second includes biological upgrading through controlled fermentation, solid-state fermentation and enzymatic treatment, where microbial or enzymatic activity modifies the matrix and may release bound compounds. The third includes extraction or concentration of selected bioactive fractions using conventional or green technologies. These approaches differ in cost, scalability, regulatory classification and circular value, and should not be treated as interchangeable strategies [18,57,58].

### 4.2. Drying, Milling and Physical Stabilization

Drying is one of the most common stabilization methods for agro-industrial by-products. It reduces water activity, limits microbial spoilage, decreases volume, facilitates transport and allows more precise diet formulation. At the same time, drying can affect heat- and oxygen-sensitive compounds, including polyphenols, carotenoids, tocopherols, volatile molecules and unsaturated lipids. The final effect depends on temperature, time, oxygen exposure, light and particle size [3,59]. Tomato pomace illustrates this balance between stabilization and molecular preservation. Drying improves shelf-life and facilitates subsequent use, but lycopene and β-carotene are sensitive to heat, oxygen, and light. Studies on tomato pomace valorization have shown that drying conditions influence carotenoid recovery and stability; therefore, the usefulness of dried tomato pomace depends on the compromise between moisture reduction, carotenoid preservation, bioaccessibility and processing cost [40,59]. Milling and particle-size reduction can improve diet homogeneity and increase the contact surface between digestive enzymes, ruminal microorganisms and plant tissues, thereby potentially enhancing digestibility and the release of nutrients and bioactive compounds from by-products. However, excessive grinding may alter ruminal fermentation, reduce physically effective fibre in ruminant diets, accelerate oxidation in lipid-rich materials and modify palatability. Physical processing should therefore be adapted to species, diet type and experimental objective rather than applied as a generic upgrading step [7,31]. Dried grape pomace provides a useful example of how physical processing can reshape the value of a by-product. Micronization and air-classification protocols can modify nutritional traits, polyphenol distribution and in vitro digestion behavior, suggesting that winery residues can be tailored for specific feeding purposes instead of being treated as uniform material [38]. Pelleting and extrusion may also improve handling, reduce feed sorting and increase diet uniformity, especially in poultry, pigs and aquaculture. However, pressure and heat may reduce heat-sensitive bioactives or accelerate lipid oxidation when residues are rich in unsaturated fatty acids. Studies using pelleted, extruded or thermally treated by-products should therefore report the composition of the final processed ingredient as administered to animals, not only the composition of the raw residue [3,7].

### 4.3. Ensiling and Co-Ensiling of Moist By-Products

Ensiling is one of the most practical options for stabilizing moist agro-industrial by-products in animal feeding systems. It relies on anaerobic fermentation, mainly driven by lactic acid bacteria, and can reduce the need for energy-intensive drying. This makes it attractive for fruit and vegetable residues, tomato pomace, citrus pulp, grape pomace and mixed local by-product streams, particularly in ruminant systems where moist and fibrous ingredients can be more easily included than in monogastric diets [7,33].

The main advantage of ensiling is that the whole matrix is fermented and preserved. Nutrients, fibres, residual lipids and bioactive compounds remain together, allowing the by-product to act as a feed ingredient rather than only as a source of extracted molecules. In lambs, silages produced from carrot, sweet potato, potato residues and tomato pomace have been used to replace part of a concentrate-based diet, supporting the practical value of local residues when stabilization is properly controlled [33]. Grape pomace also shows how ensiling can modify nutritional and functional traits before animal feeding. Ensiling Nero di Troia grape pomace, particularly with Lactiplantibacillus plantarum 5BG, improved in vitro digestibility and modified phenolic profile, microbial quality and rumen fermentation traits, although this evidence remains mechanistic because it does not directly demonstrate improved animal-derived food quality [60].

Co-ensiling can improve the feasibility of residues that are too wet, poor in fermentable sugars, rich in fat or unsuitable for ensiling alone. Combining complementary materials can improve dry matter content, buffering capacity and fermentation quality, but it also produces a new composite ingredient. Co-ensiled materials should therefore be described through their own chemical, microbiological and nutritional profile [7,33]. In pigs, silages based on olive oil, winery and cheese-industry by-products have also been evaluated as circular feed ingredients, with attention to growth, health, gut microbiota and meat quality, suggesting that controlled mixed-residue silages may be useful beyond ruminant systems when inclusion levels are realistic, and the final product is assessed [34,53].

Ensiling should not be presented as automatically beneficial. Fermentation quality depends on moisture, soluble carbohydrates, buffering capacity, particle size, contamination level and the epiphytic microbial population of the starting material. Poor ensiling can lead to nutrient losses, undesirable fermentation products, spoilage microorganisms and reduced palatability. Lactic fermentation may modify polyphenols, organic acids, soluble carbohydrates and volatile molecules, but the direction and magnitude of these changes are substrate-dependent and should be measured directly rather than assumed [3,18].

### 4.4. Solid-State Fermentation and Enzymatic Upgrading

Solid-state fermentation is a promising strategy for upgrading agro-industrial residues. It involves the growth of selected microorganisms on moist solid substrates with little or no free water, and is suitable for cereal brans, brewer’s spent grain, oilseed cakes, fruit peels, pomaces and other lignocellulosic matrices. Filamentous fungi, yeasts and lactic acid bacteria can produce cellulases, xylanases, pectinases, β-glucosidases, tannases and feruloyl esterases, which may degrade the plant matrix and release compounds bound to cell-wall structures [18,61,62]. This effect is particularly relevant for cereal-derived residues and brewer’s spent grain, where many phenolic acids are linked to arabinoxylans and other cell-wall polymers. Fermentation can increase the proportion of free phenolic acids, improve antioxidant activity and generate smaller microbial metabolites. In brewer’s spent grain, solid-state fermentation enhanced the release of polyphenols in UPLC-MS-based analyses, although the response depended on microbial strain, fermentation time, substrate composition and processing conditions [31,63]. The main limitation of solid-state fermentation is standardization. Laboratory improvements in antioxidant capacity, polyphenol release or protein hydrolysis do not necessarily translate into consistent animal responses or industrial feasibility. The final ingredient depends on substrate composition, inoculum, microbial strain, moisture, aeration, temperature, fermentation time, and post-fermentation drying. Microbial safety also needs control, especially when fungi or mixed microbial cultures are used. Fermented by-products should therefore be treated as new ingredients rather than simply as improved versions of the original residue [62,64]. Enzymatic treatment offers a more targeted alternative. Exogenous cellulases, xylanases, pectinases, proteases and esterases can degrade structural carbohydrates, release bound polyphenols, improve protein digestibility and reduce viscosity or anti-nutritional constraints. Compared with fermentation, enzymatic treatment may be more controllable, but it can also be more expensive and less compatible with low-cost circular feeding if enzymes, processing time and drying steps increase the overall cost of the ingredient. Its usefulness should therefore be judged against measurable improvements in bioaccessibility, digestibility, animal response and final product quality [29,61,65].

### 4.5. Green Extraction, Concentrated Fractions and the Whole-Matrix/Extract Distinction

Green extraction technologies are increasingly used to recover bioactive compounds from agro-industrial by-products. Ultrasound-assisted extraction, microwave-assisted extraction, pressurized liquid extraction, subcritical water extraction, supercritical fluid extraction and natural deep eutectic solvents have been proposed as alternatives to conventional solvent extraction. These approaches may reduce extraction time, solvent use and energy demand while improving the recovery of polyphenols, carotenoids, terpenes, oils and other functional molecules [57,58,66]. However, extraction yield alone does not define sustainability or feed applicability. Solvent recovery, toxicity, downstream purification, regulatory acceptance and compatibility with animal feeding must also be considered before extracted fractions can be proposed as functional feed ingredients or additives for animal diets [58,67]. Tomato pomace provides a useful example, because sustainable drying combined with green deep eutectic extraction has been proposed to recover lycopene and β-carotene from this residue [59]. A key distinction should be made between whole-matrix feeding and extract-based strategies. If the whole dried pomace or residue is included in the diet, it remains a circular feed ingredient that contributes fibre, residual lipids, bioactive compounds and minor nutrients. If a carotenoid- or polyphenol-rich fraction is extracted and concentrated, the intervention becomes closer to a standardized bioactive source derived from a by-product. Whole-matrix feeding is generally more coherent with nutrient recovery and feed replacement, whereas extracts or concentrated fractions offer greater molecular precision but may require solvents, filtration, concentration, drying and quality-control steps. Both approaches can be valid, but they answer different questions and should be evaluated with different criteria [7,9,57,58].

### 4.6. Processing, Safety and Sustainability Should Be Evaluated Together

Processing can improve the functional value of agro-industrial by-products, but it can also introduce new risks. Drying limits microbial spoilage but does not remove chemical contaminants. Ensiling stabilizes moist residues but cannot correct poor raw-material quality. Fermentation may reduce some anti-nutritional factors but requires microbial control. Extraction can concentrate beneficial compounds together with unwanted residues if selectivity is poor [3,18]. The final processed ingredient should therefore be characterized after processing. At minimum, this characterization should include dry matter, proximate composition, fibre fractions, lipid profile, lipid oxidation indicators, key bioactive compounds and microbial quality. Depending on the matrix and processing method, fermentation acids, mycotoxins, pesticide residues, heavy metals or solvent residues may also need to be assessed. A residue cannot be considered circular or functional if its use transfers chemical or microbiological hazards along the feed–animal–food continuum [3,7]. The environmental dimension also depends on processing intensity. A strategy that increases antioxidant capacity, digestibility or extraction yield may lose part of its circular advantage if it requires high energy input, long transport, expensive purification or complex waste management. The most convincing approaches are those that preserve or improve the bioactive fraction, maintain feed safety, reduce variability, remain economically feasible and produce measurable effects in animal-derived foods without excessive processing intensity [57,58,68].

## 5. Microbiome-Mediated Biotransformation of By-Product-Derived Bioactives

### 5.1. The Microbiome as a Biochemical Filter Between Feed and Food

The biological effect of agro-industrial by-products is strongly shaped by the gastrointestinal microbiome. This is most evident in ruminants, where the rumen is the first major site of microbial transformation, but it is also relevant in pigs, poultry, rabbits and fish, where intestinal microbiota can modify fibres, oligosaccharides, polyphenols and other dietary compounds before absorption or excretion. The molecules present in a by-product are therefore not necessarily the same molecules that reach blood, milk, muscle, adipose tissue or egg yolk [11,13]. The microbiome can release compounds from the plant matrix, degrade native molecules, generate smaller metabolites, alter fermentation patterns and change nutrient availability for the host. A polyphenol-rich grape pomace, an olive residue rich in hydroxytyrosol derivatives, a citrus pulp rich in flavanones or a cereal bran rich in fibre-bound phenolic acids may therefore produce different responses according to the microbial ecosystem that processes it. This helps explain why the same by-product may behave differently across species, animals, diets and production systems [11,13]. This microbial step is central for interpreting functional animal-derived foods. If a dietary compound is degraded before absorption, the absence of the native molecule in milk, meat or eggs does not necessarily mean that the by-product had no biological effect. The relevant intermediates may be microbial metabolites, host-conjugated forms, or indirect changes in fermentation, lipid metabolism, oxidative balance or immune response. Multi-omics work in dairy cows has shown that rumen microbiome, rumen metabolome and host metabolome contribute together to individual production phenotypes, supporting the idea that microbial metabolism is not only a consequence of diet but a determinant of animal response [69].

### 5.2. Rumen Transformation of Polyphenols and Plant Secondary Metabolites

In ruminants, the rumen is the main site whereby-product-derived bioactives are released, degraded or transformed before intestinal absorption. Its microbial ecosystem includes bacteria, archaea, protozoa and anaerobic fungi involved in fibre degradation, protein metabolism, lipid biohydrogenation and methane formation. When polyphenol-rich by-products enter this system, they can interact with microbial enzymes and cell structures, alter microbial populations and generate metabolites that differ from the original dietary compounds [13,70]. Several reactions are relevant for polyphenol metabolism. Glycosidases remove sugar moieties from flavonoid glycosides, esterases release phenolic acids bound to cell-wall polysaccharides, tannases hydrolyse galloylated compounds, and reductases, decarboxylases, dehydroxylases and ring-cleavage enzymes convert complex polyphenols into smaller aromatic acids. These reactions may increase bioaccessibility, but they can also reduce the concentration of the parent compound and make feed-to-product tracing difficult when only native molecules are measured [11]. This is relevant for grape pomace, olive residues, pomegranate peel and citrus pulp. Condensed tannins, anthocyanins, flavanones, secoiridoid derivatives and ellagitannins do not behave as a single group in the rumen. Some compounds bind proteins and modify nitrogen metabolism, some are degraded into lower-molecular-weight metabolites, some affect microbial lipid metabolism, and others remain poorly available because they are associated with the fibre matrix. Total phenolic content is therefore a weak predictor of ruminal fate [13,19,20]. Ellagitannin metabolism provides a useful example of microbial specificity. Ellagitannins and ellagic acid, present in pomegranate and other fruit residues, are poorly absorbed as native compounds and can be converted by gut bacteria into urolithins. These metabolites differ in biological activity and are produced according to individual metabotypes, a concept well established in human nutrition and relevant for interpreting variable animal responses to polyphenol-rich feeds [27,71]. Isoflavone metabolism follows a similar principle. Equol is produced from daidzein by specific bacterial populations and can be detected in milk when suitable precursors are supplied, and the microbial ecosystem supports conversion [72,73]. These examples show that functional enrichment may depend on microbial capacity as much as on dietary supply.

### 5.3. Rumen Fermentation, Methane and Nitrogen Metabolism

By-products rich in tannins, flavonoids, pectins and structural carbohydrates can modify rumen fermentation, but the response is not always straightforward. Changes in volatile fatty acid profile, ammonia concentration, microbial protein synthesis, methane production and fibre degradation may derive from direct microbial effects, altered substrate availability, protein binding or changes in passage rate. Fermentation outcomes should therefore be read together with digestibility, intake and animal performance [1,13,70]. Tannins are particularly relevant because they can bind dietary proteins and reduce ruminal protein degradation, potentially increasing the flow of undegraded protein to the intestine. Under appropriate conditions, this may improve nitrogen-use efficiency and reduce ruminal ammonia formation. At higher doses, however, tannins can depress microbial activity, fibre digestion and feed intake, especially when highly reactive fractions are used or when the basal diet is already limiting in fermentable energy or degradable nitrogen. This dual behavior makes tannin-rich by-products useful, but difficult to standardize [13,70]. Methane mitigation requires the same caution. Some tannin- or phenolic-rich by-products may reduce methane by inhibiting methanogens, reducing protozoa-associated methanogenesis or redirecting hydrogen toward alternative sinks. In other cases, lower methane may simply reflect reduced fibre degradation or lower intake, which is less desirable from a production perspective. Methane output should therefore be evaluated together with digestibility, fermentation efficiency, milk or meat production and nutrient utilization [1,13,70]. In vitro evidence on ensiled Nero di Troia grape pomace shows that microbial stabilization can modify the interaction between a winery by-product and rumen fermentation, especially when *Lactiplantibacillus plantarum* 5BG is used. However, because this evidence does not include an in vivo feeding response or animal-derived food quality, it should be considered mechanistic rather than proof of functional product improvement [60]. Similarly, grape, citrus, tomato and hazelnut residues rich in polyphenols have been shown to reduce methane production in sheep rumen fermentation systems, although responses differed among by-products and were associated with changes in degradability and fermentation variables. This confirms that methane reduction depends on the specific matrix and its interaction with the rumen ecosystem, not simply on total polyphenol content [1].

### 5.4. Microbiome-Mediated Effects on Lipid Biohydrogenation

Ruminal lipid biohydrogenation is one of the clearest links between by-products, microbiome, and animal-derived food quality. In ruminants, dietary unsaturated fatty acids are extensively transformed by rumen microorganisms before absorption, and this process strongly affects the fatty acid profile of milk and meat [74]. By-products can influence this pathway by supplying unsaturated lipids, modifying microbial populations involved in biohydrogenation, or providing polyphenols and tannins that interfere with specific microbial steps [13,14]. Tannins and other polyphenol classes may modulate bacteria involved in the conversion of linoleic and α-linolenic acids into more saturated end-products. This can increase the intestinal flow of biohydrogenation intermediates such as vaccenic and rumenic acids, with potential effects on milk and meat fatty acid profiles [75]. A meta-analysis on dietary tannins confirmed their capacity to modulate rumen fermentation, methane emissions and polyunsaturated fatty acid biohydrogenation, although responses depend on tannin type, dose and diet composition [14]. This mechanism is often used to explain the effects of grape pomace, olive residues, pomegranate by-products and tomato pomace on milk and meat fatty acids. It should not be assumed, however, when ruminal fermentation, microbial markers or biohydrogenation intermediates have not been measured. A more favorable product fatty acid profile may also result from direct lipid supply, changes in dry matter intake, replacement of starch or fibre in the basal diet, or altered mammary and tissue lipid metabolism [6,13,14]. This point matters for functional-food interpretation. An increase in unsaturated fatty acids, vaccenic acid or rumenic acid in milk or meat is valuable, but it should not automatically be attributed to the antioxidant activity of polyphenols. The by-product may act through lipid supply, microbial modulation, energy replacement or a combination of these mechanisms. Stronger evidence requires the joint assessment of the by-product lipid fraction, rumen fermentation, microbial taxa or functions related to biohydrogenation, plasma lipid intermediates and final product composition [14,16].

### 5.5. Intestinal Biotransformation in Monogastric Species

In monogastric animals, by-product bioactives bypass the rumen but are still exposed to microbial transformation in the gastrointestinal tract, especially in the hindgut. Pigs and poultry have a lower fermentative capacity than ruminants, but microbial enzymes can still transform polyphenols, fibres and oligosaccharides into smaller metabolites, short-chain fatty acids and other bioactive compounds [76]. Polyphenol metabolism includes deglycosylation, de-esterification, demethylation, dehydroxylation, decarboxylation and aromatic ring cleavage. These reactions can produce phenylacetic, phenylpropionic, hydroxybenzoic and hydroxycinnamic derivatives, which may be more absorbable than the original molecules. After absorption, these metabolites can undergo host conjugation, mainly glucuronidation, sulphation and methylation, before reaching tissues or being excreted [11]. Thus, the compounds that may influence meat or eggs are often microbial-host co-metabolites rather than native by-product molecules. By-products rich in fermentable fibres may also influence gut ecology through short-chain fatty acid production. Pectins from citrus and apple residues, arabinoxylans from cereal brans and other soluble or partially fermentable polysaccharides can support microbial fermentation and increase acetate, propionate or butyrate production, depending on substrate and animal species [3,4]. These metabolites can influence gut barrier function, immune tone and energy metabolism. However, the link between gut fermentation and food quality remains weak in many livestock studies. A favorable change in gut microbiota does not automatically imply improved meat, milk or egg quality. In finishing pigs, mixed agro-industrial by-product silages have been reported to modify intestinal microbiota, increasing beneficial lactic acid bacteria and reducing potentially undesirable bacterial groups, while also affecting some meat-quality traits. Such studies are useful because they connect circular feeding, microbiota and product quality within the same experimental framework [34,53].

**Figure 3:**
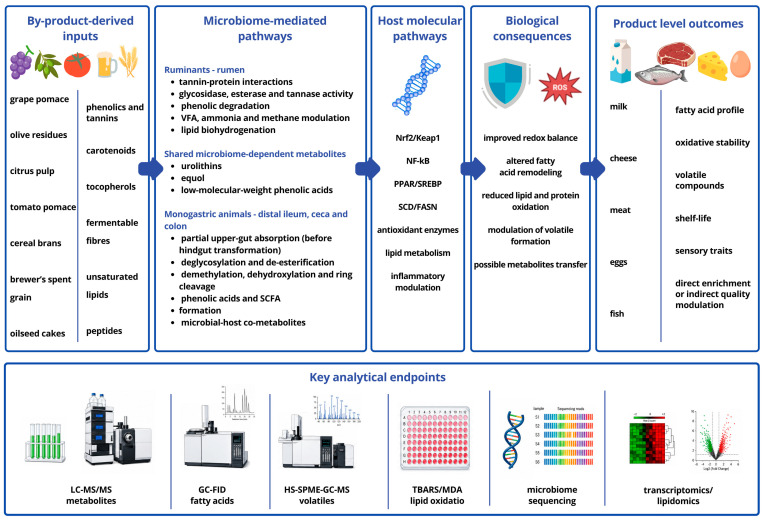
Main microbiome-mediated and host molecular pathways through which agro-industrial by-products may modulate the nutritional, oxidative and technological quality of animal-derived foods.

Although several microbial processes occur in both ruminants and monogastric animals, their sites of occurrence, timing relative to nutrient absorption, and overall metabolic significance differ considerably. In ruminants, extensive microbial transformation takes place in the rumen before small-intestinal absorption. These processes include tannin–protein interactions, microbial hydrolysis, aromatic ring cleavage, fermentation, nitrogen metabolism and lipid biohydrogenation. In monogastric species, some native compounds or their aglycones may be released and absorbed in the upper gastrointestinal tract. In contrast, poorly absorbed tannins, flavonoid glycosides and fibre-bound phenolic compounds reach the distal intestine, where they undergo further microbial transformation, including deglycosylation, de-esterification, demethylation, dehydroxylation, decarboxylation and ring cleavage. Consequently, the effects observed in ruminant-derived products are strongly influenced by pre-absorptive ruminal fermentation and lipid biohydrogenation. In monogastric animals, these effects more often result from the combined contribution of direct absorption or tissue deposition of dietary compounds and the formation of microbial–host co-metabolites in the hindgut. The key differences between these digestive systems are summarized in Supplementary Table S3 and conceptually integrated in Figure 3.

### 5.6. Microbial Metabolites and Product-Level Interpretation

Microbial metabolites are important because they can provide a closer link between by-product intake and final product quality than broad microbiome descriptors alone. For polyphenol-rich by-products, relevant metabolites may include phenolic acids, hydroxytyrosol and tyrosol derivatives, equol, urolithins and other low-molecular-weight compounds produced through microbial and host metabolism [11,27,72]. This distinction is important when interpreting whether a by-product supports direct enrichment, metabolite-mediated transfer or indirect modulation of product quality. A dairy product containing hydroxytyrosol, tyrosol or related metabolites after olive-derived supplementation provides stronger evidence of compound transfer than a product showing only higher antioxidant capacity. Similarly, equol or urolithin production indicates that microbial transformation has occurred, but it does not automatically prove that the final food has a functional effect for consumers. The analytical target should match the claim: native compounds for direct enrichment, microbial or host-derived metabolites for biotransformation, and oxidative, lipid or sensory endpoints for indirect quality modulation [21,22,27]. Metabolomics can help identify these intermediates, but it should not be used only as a descriptive fingerprint. A list of differentially abundant metabolites becomes biologically stronger when linked to microbial functions, host markers, and measurable changes in milk, meat, eggs, or fish [77]. Detecting polyphenol metabolites in milk or cheese supports compound transfer; detecting changes in rumen or serum metabolites without final product data supports a mechanistic hypothesis. This difference should be reflected in the strength of the claim [22,69].

### 5.7. Limitations and Implications for Functional Animal-Derived Foods

Despite strong biological plausibility, the evidence that microbiome-mediated transformation of by-product bioactives directly improves animal-derived foods remains incomplete. Many studies measure animal performance and product quality but not the microbiome, whereas others describe rumen or gut microbiota without evaluating milk, meat, eggs or derived products. Some studies report 16S rRNA sequencing data, but without metabolomics, microbial function or targeted metabolites. The pathway from by-product to microbiome, host metabolism and final food quality is therefore often reconstructed rather than directly demonstrated [13,69]. Broad taxonomic descriptors are another limitation. Changes in alpha diversity, beta diversity or the relative abundance of large microbial groups can be informative, but they rarely explain biological function on their own. For polyphenol metabolism, fibre fermentation, methane formation or lipid biohydrogenation, functional genes, enzymes and metabolites may be more useful than general shifts in microbiota composition [13,14]. Temporal resolution and individual variability also deserve attention. Microbial adaptation to a new by-product may require days or weeks, especially when the ingredient contains tannins, fermentable fibres, residual lipids or fermentation products. Short trials may capture acute changes but miss adaptation, whereas long trials with only one sampling point may fail to describe transformation dynamics. Animals receiving the same by-product may also produce different microbial metabolites because of differences in their initial gut microbiome, diet history, age, physiological stage, health status and production level. This concept is well known for human polyphenol metabotypes, such as urolithin and equol production, and may also be relevant in livestock, although it has been less systematically investigated [11,27,69,72]. For functional animal-derived foods, these limitations have practical consequences. Microbiome-mediated biotransformation helps explain why agro-industrial by-products are promising, but also why their effects are difficult to predict. The strongest studies will be those that follow the chain from feed matrix to microbiome, microbial metabolites, host response, and final product quality—these all measured within the same experimental study. However, achieving the latter is not easy but doable, as many experiments do not set objectives to cover all the measurements, as it is laborious and expensive to undertake. Such integrated evidence is needed to distinguish a circular feed ingredient with general nutritional value from a by-product able to generate reproducible functional effects in milk, cheese, meat, eggs, fish or derived products [9,69].

## 6. Transfer of Bioactive Compounds and Metabolites to Animal-Derived Foods

### 6.1. Transfer Is Not a Single Biological Process

The transfer of by-product-derived compounds to animal-derived foods is often described as a direct movement from feed to milk, meat, eggs or fish tissues. In practice, the process is more selective. Digestion, microbial metabolism, absorption, host conjugation, hepatic transformation, tissue uptake, mammary secretion, yolk deposition and muscle incorporation can all modify the molecules originally present in the feed matrix [11]. Three situations should be separated. The first is direct or relatively direct enrichment, where native molecules or closely related compounds can be quantified in the final product. This is more likely for lipophilic molecules, such as carotenoids and tocopherols, especially in egg yolk or fat-rich tissues. The second is metabolite transfer, where the compounds detected in the product are microbial or host-derived forms, such as hydroxytyrosol or tyrosol conjugates, phenolic acids, equol or urolithins. The third is indirect quality modulation, where no specific dietary molecule is clearly deposited, but the by-product modifies rumen biohydrogenation, oxidative stability, volatile formation, inflammation or lipid metabolism [11,72]. These mechanisms differ in evidential strength. A cheese containing hydroxytyrosol or tyrosol metabolites after supplementation with olive-derived polyphenols provides stronger evidence of molecular transfer than a meat sample showing lower lipid oxidation after a polyphenol-rich diet without metabolite tracing. Both outcomes may be valuable, but they do not support the same type of claim. Similarly, a more favourable milk fatty acid profile after feeding grape, olive or tomato by-products may reflect ruminal biohydrogenation, lipid supply or mammary lipid metabolism rather than direct deposition of plant polyphenols in milk [6,14,21]. For this reason, product interpretation should specify whether the evidence supports direct enrichment, metabolite-mediated transfer or indirect quality modulation. This distinction is especially useful when comparing ruminant products, where microbial transformation is extensive, with eggs, where carotenoid deposition can be more direct and easier to verify [21,25].

### 6.2. Milk and Dairy Products

Milk is a useful matrix for evaluating dietary effects because it is produced continuously and contains both aqueous and lipid fractions. At the same time, it is a complex secretion. The appearance of a dietary compound in milk depends on ruminal transformation, intestinal absorption, host conjugation, mammary transport, and dilution into milk compartments. This is especially relevant for polyphenols, which rarely reach milk or dairy products as unchanged native molecules [11]. Olive-derived polyphenols provide one of the clearest examples of measurable transfer to dairy products. In dairy sheep, supplementation with olive mill wastewater rich in polyphenols increased hydroxytyrosol, tyrosol and their sulphate metabolites in cheese, showing that selected by-product-derived metabolites can be detected in the final product when targeted methods are used [21]. Further evidence from Italian cheeses confirmed hydroxytyrosol, tyrosol and related metabolites as traceable molecules in dairy matrices, supporting a chemically defined form of dairy enrichment from olive-derived sources [22]. Citrus pulp represents a different case. In dairy cows, the inclusion of pelleted citrus pulp increased total phenolic acids, flavonoids, and ferric-reducing antioxidant power in milk [23]. This suggests an effect on antioxidant-related traits, but the underlying molecules were less clearly defined than in studies targeting olive polyphenol metabolites. The result is still relevant, but it should be described as antioxidant-related modulation unless specific compounds or metabolites are quantified in the final product. For milk fat, by-products rich in phenolic acids, tannins, or residual lipids may alter the fatty acid profile through changes in rumen biohydrogenation and lipid supply. Meta-analytic evidence in dairy sheep and goats indicates that polyphenol-rich agro-industrial by-products can reduce saturated fatty acids and increase nutritionally relevant unsaturated fatty acids, including vaccenic, rumenic and linolenic acids [16]. Similar effects have been reported for grape, pomegranate, olive and tomato by-products in dairy ruminants, without necessarily depressing milk production [6]. These changes are important for nutritional quality, but they represent lipid-profile modulation rather than direct polyphenol enrichment. Cheese adds another level of complexity. Milk composition, microbial ecology, ripening conditions, proteolysis, lipolysis and volatile formation can all affect the final product. By-product feeding studies targeting cheese should therefore evaluate not only raw milk composition, but also cheese-making performance, ripening evolution, polyphenol metabolites, fatty acid profile, volatile compounds, oxidative stability and sensory traits as a whole or in combinations [21,22,78].

### 6.3. Meat and Meat Products

In meat, dietary by-products can influence meat quality, nutritional value and consumer choice through lipid composition, oxidative stability, color stability, volatile formation and, less frequently, direct deposition of dietary metabolites. The most consistent evidence concerns oxidative stability and fatty acid profile. Polyphenol-rich residues may reduce lipid oxidation during storage or processing, whereas lipid-rich residues and by-products may modify the proportion of saturated, monounsaturated, polyunsaturated fatty acids and their intermediates. These effects can improve nutritional or functional quality, but they also need careful interpretation [9,79,80]. Lower lipid oxidation after feeding grape pomace, olive residues or other antioxidant-rich matrices does not necessarily prove deposition of native polyphenols or their metabolites in muscle. It may reflect improved systemic antioxidant status, changes in fatty acid profile, reduced oxidative pressure, or interactions between dietary antioxidants and endogenous defense systems [75]. Studies that combine TBARS or other oxidation markers with polyphenol metabolite tracing, fatty acid profile, color stability, and sensory data provide stronger evidence than studies based on a single oxidative endpoint [9,51]. The balance between fatty acid improvement and oxidative stability is particularly important. Increasing unsaturated fatty acids can improve the nutritional profile of meat, but it may also increase susceptibility to oxidation if antioxidant protection is insufficient [44,45]. By-products that supply both unsaturated lipids and antioxidant molecules, such as grape seeds, tomato seeds, olive residues or oilseed cakes, may be valuable, but their effects should be evaluated through fatty acid profile, lipid oxidation, color stability, volatile compounds and sensory traits together [6,79]. Recent work on artichoke bracts silage in beef finishing diets provides a useful example of a whole-matrix approach, because the by-product was evaluated not only as a feed ingredient but also in relation to meat quality during dry aging [56,81,82]. This kind of design is relevant for circular functional foods because it connects local residue use, animal feeding and a value-adding meat process. Still, product-quality improvement should be kept distinct from direct cellular enrichment unless specific bioactive compounds or metabolites are measured in the edible tissue [83].

### 6.4. Eggs

Eggs are one of the clearest animal-derived products for studying feed-to-food enrichment, especially for carotenoids. The yolk is a lipid-rich matrix where pigments and lipophilic components can be deposited directly and measured with relative precision. Tomato-derived by-products are therefore particularly relevant because they contain lycopene, β-carotene, tocopherols, seed oil, and other minor compounds [25,26]. Tomato powder supplementation in laying hens increased yolk carotenoids and vitamin A and reduced yolk lipid peroxidation, providing a clear example of carotenoid-rich feed producing measurable changes in the final product [25]. Meta-analytic evidence also supports the practical potential of tomato waste in laying hens, with reported improvements in performance and egg-quality traits, although responses depend on inclusion level, drying method, tomato matrix and basal diet formulation [41]. More broadly, natural carotenoid supplementation has been associated with changes in yolk pigmentation, egg quality and selected immune-related outcomes [42]. Eggs can also reflect changes in dietary fatty acids. Oilseed cakes, orchard by-products, seed-rich residues and other lipid-containing by-products may modify yolk fatty acid composition, antioxidant levels, vitamins and minerals [84]. The functional value of this enrichment depends on oxidative stability, because increasing polyunsaturated fatty acids may also increase susceptibility to lipid oxidation, unless adequate antioxidant protection is maintained. Egg-enrichment studies should therefore evaluate fatty acid profile, carotenoid or tocopherol deposition, lipid oxidation and sensory acceptability together [26,42]. Polyphenol-rich by-products may improve antioxidant status or reduce yolk oxidation, but the evidence for direct polyphenol deposition in eggs is less straightforward than for carotenoids. When grape pomace, olive residues or other polyphenol matrices are used in laying hens, studies should distinguish among improved antioxidant status of the bird, reduced yolk oxidation and actual transfer of polyphenol metabolites to the egg. These outcomes are related, but they are not equivalent [11,19].

### 6.5. Fish and Aquaculture Products

Aquaculture is increasingly relevant for by-product valorization because aquafeeds require sustainable alternatives to conventional resources, and fish fillets are highly sensitive to lipid oxidation because of their high content of unsaturated fatty acids. Fruit and vegetable processing by-products, grape pomace, olive-derived fractions and plant antioxidant sources have therefore been investigated as functional feed ingredients or additives in fish diets. Responses, however, depend strongly on fish species, digestive physiology, lipid metabolism and tolerance to plant-derived residues [85,86]. Grape pomace has recently been evaluated in European sea bass diets with attention to oxidative status, intestinal microbiota and fillet quality. The inclusion of grape pomace in the diet improved antioxidant-related responses, helped prevent oxidation during storage and affected physiological, immunological and fillet-quality traits [54]. This is a useful example because it links a polyphenol-rich by-product with gut-related responses and edible fillet characteristics. In common carp, dietary grape pomace at 5% and 10% has also been evaluated for growth, physiological parameters and biochemical composition, supporting wider interest in grape-derived residues for aquafeeds [55]. By-product-derived lipid sources are another important issue in aquaculture. Replacing fish oil with acid oils or other circular lipid sources may affect fillet fatty acid profile, oxidative stability and sensory acceptability. In European sea bass, dietary acid oils used as partial substitutes for fish oil influenced fillet lipid composition and quality, showing that circular lipid ingredients must be evaluated against the nutritional and sensory quality of the final seafood product [87]. Overall, aquaculture broadens the relevance of agro-industrial by-products but also adds complexity. Fish fillets are excellent matrices for studying oxidative stability and fatty acid composition, but plant residues may affect feed intake, digestibility, gut health and product flavor differently across species. By-product use in aquafeeds should therefore be supported by fillet fatty acid profile, oxidation indices, sensory evaluation and shelf-life data, not only by growth or feed-conversion results [54,85,86].

### 6.6. Analytical Requirements for Demonstrating Transfer and Functionality

Demonstrating compound transfer, metabolite-mediated transformation, or functional improvement requires endpoints that match the proposed mechanism. When the objective is direct enrichment, the target compound or metabolite should be quantified in the final product with validated methods. Carotenoids in egg yolk, hydroxytyrosol and tyrosol metabolites in cheese, equol in milk or specific lipid classes in meat and fish need targeted analytical approaches rather than broad antioxidant assays alone [21,22,26]. When the expected effect is indirect quality modulation, the analytical design should capture the relevant product trait. For lipid-related claims, fatty acid profile should be combined with lipid oxidation markers and, when possible, lipidomics. For oxidative stability, TBARS or peroxide values should be supported by additional markers such as protein oxidation, color stability, volatile compounds and shelf-life. For aroma-related claims, volatile profiling should be interpreted together with sensory analysis. For dairy products, cheese-making performance and ripening traits may be as important as raw milk composition [9,48,79]. A second requirement is adequate feed characterization. Product-level changes are difficult to interpret when the by-product is described only by name or inclusion level. The processed material used in the diet should be characterized for the nutrient and bioactive fractions relevant to the expected effect, such as phenolic acids, tannins, carotenoids, tocopherols, lipid profile, oxidation status, fermentability and safety parameters [3,7]. Finally, analytical interpretation should avoid overstating the claim. A higher antioxidant capacity in milk, lower TBARS in meat or improved pigmentation in eggs can be valuable, but these outcomes do not automatically demonstrate a functional food in the strict sense. Stronger evidence comes from studies that combine by-product characterization, realistic feeding design, biological intermediates and direct measurements in the final animal-derived product. This approach allows direct enrichment, metabolite-mediated transformation and indirect quality modulation to be distinguished with greater confidence.

## 7. Host Molecular Pathways Linking By-Product Feeding and Product Quality

### 7.1. From Dietary Bioactives to Host Response

The effects of agro-industrial by-products on animal-derived foods are not explained only by the transformation of nutrients and bioactives from feed to animal products. In many cases, the relevant response occurs after digestion, microbial transformation and absorption, when dietary compounds or their metabolites interact with host metabolism. These interactions may involve oxidative balance, inflammatory response, lipid metabolism, immune status, mitochondrial function and nutrient partitioning, all of which can influence the composition and stability of milk, meat, eggs and fish tissues [9,88]. This is particularly relevant for polyphenol-rich matrices. Native polyphenols are often transformed before reaching systemic circulation, and their metabolites may act at concentrations much lower than those used in many in vitro models. Their biological effect depends on bioavailability, tissue exposure, redox status and the metabolic state of the animal. A dietary by-product should therefore not be expected to act as a simple antioxidant additive; rather, its effects may be mediated through changes in cellular signaling, microbial metabolites, lipid metabolism and product chemistry [9,11]. For functional animal-derived foods, host pathways are useful only when they help explain measurable product traits. Higher antioxidant enzyme activity, lower inflammatory response, or altered lipid- mediated gene expression are informative, but they become stronger evidence when linked with the functional characteristics of end products such as milk fatty acid profile, meat oxidative stability, yolk pigmentation, cheese volatile compounds or fish fillet shelf-life. This section therefore focuses on host mechanisms that can plausibly connect by-product feeding with product-level outcomes.

### 7.2. Oxidative-Stress Pathways and Redox-Sensitive Signaling

Oxidative balance is one of the most frequently proposed mechanisms linking bioactive by-products with product quality. Polyphenols, carotenoids, tocopherols, and other antioxidant-related substances may influence redox status directly or indirectly, but their in vivo action is rarely limited to chemical radical scavenging. More often, they interact with endogenous antioxidant systems, mitochondrial function and redox-sensitive signaling pathways [89,90]. The Nrf2 pathway is central in this context because it regulates genes involved in antioxidant defense, detoxification, and cellular protection. Some dietary phytochemicals can activate Nrf2-related responses, increasing the expression of antioxidant enzymes and cytoprotective proteins. At the same time, Nrf2 does not act in isolation. It interacts with inflammatory signaling, cellular stress responses and metabolic regulation, which makes it relevant for interpreting the effects of complex by-product matrices rather than purified compounds alone [91,92]. This pathway is relevant for animal-derived foods because oxidative status affects product stability. In meat and fish, lipid and protein oxidation influence color, flavor, texture, drip loss and shelf-life. In milk and eggs, oxidative balance may affect lipid stability, antioxidant capacity and storage behaviour. By-products rich in polyphenols or carotenoids may help reduce oxidative deterioration, but product-level endpoints remain essential. An increase in antioxidant enzymes or plasma antioxidant capacity is useful mechanistic evidence, but it does not prove improvement of the final food unless oxidation markers, color stability, volatile compounds or shelf-life are also measured [9,79,93].

### 7.3. Inflammatory Signaling and Immune-Metabolic Tone

Inflammatory signaling is closely connected with oxidative balance and metabolism. Pathways such as NF-κB respond to oxidative stress, microbial products, cytokines, and metabolic challenges, and can influence tissue function, immune status, and nutrient partitioning. Dietary phytochemicals may modulate these pathways, but the response depends on dose, metabolite availability, animal physiology, and basal inflammatory status [89,90,94]. For by-product feeding studies, this mechanism is relevant when inflammation affects performance, health, or product quality. A reduction in systemic inflammatory responses may support better nutrient use, improved oxidative stability or more favorable tissue metabolism. In dairy animals, inflammatory and oxidative status can influence mammary function and milk composition. In meat-producing animals, chronic oxidative stress or inflammation may affect muscle metabolism before slaughter and post-mortem muscle to meat conversion. In fish, immune-metabolic responses can influence fillet quality and oxidative stability during storage. However, inflammatory markers should be interpreted cautiously. Changes in cytokines, acute-phase proteins or immune-related gene expression do not automatically imply improved product functionality. They become more meaningful when associated with animal health, growth performance, tissue metabolism and measurable traits of milk, meat, eggs or fish. By-product studies should therefore avoid treating anti-inflammatory activity as a stand-alone claim unless it is connected to biological and product-level outcomes [9,90].

### 7.4. Lipid Metabolism and Fatty Acid Remodeling

Lipid metabolism is one of the strongest links between by-product feeding and animal-derived food quality. By-products may influence fatty acid profile by supplying residual lipids, modifying rumen biohydrogenation, changing the availability of microbial intermediates, or altering host lipid synthesis and desaturation [15]. In ruminants, the final fatty acid profile of milk and meat reflects both microbial and host metabolism. Ruminal biohydrogenation determines the availability of intermediates such as vaccenic acid, whereas host desaturase activity influences the formation of rumenic acid in tissues and milk [13,14,95]. Host transcriptional pathways are also involved. Genes and regulators associated with fatty acid uptake, de novo synthesis, desaturation and lipid droplet formation help determine how absorbed substrates are incorporated into milk fat, muscle or adipose tissue. PPAR-related pathways are particularly relevant because they integrate fatty acid sensing, lipid metabolism, inflammation and energy partitioning. In dairy cow mammary epithelial cells, PPARγ has been described as a positive regulator of milk fat synthesis, and fatty acids such as acetate and palmitate may partly regulate milk fat synthesis through PPARγ-related mechanisms [96]. This matters for by-products rich in unsaturated lipids, fermentable carbohydrates, or polyphenols. These matrices may influence both substrate availability and transcriptional regulation of lipid metabolism. In meat-producing animals, host lipid metabolism determines not only nutritional quality but also oxidative susceptibility. Increasing unsaturated fatty acids can improve the nutritional profile of meat, but higher unsaturation may also accelerate oxidation during storage if antioxidant protection is weak. Conversely, polyphenol- or carotenoid-rich residues may improve oxidative stability without strongly changing fatty acid composition. Fatty acid remodeling, antioxidant defense and shelf-life should therefore be evaluated together when by-products are proposed as tools for improving meat, milk, egg or fish quality [88,97,98].

### 7.5. Protein Oxidation, Proteolysis and Technological Quality

Protein oxidation is less frequently considered than lipid oxidation, but it is highly relevant for product quality. Oxidative modifications of muscle proteins can affect solubility, enzyme activity, proteolysis, water-holding capacity, tenderness, color stability and the technological performance of meat and processed products. In meat and fish, lipid and protein oxidation often occur together during storage, processing or cooking. A feeding strategy proposed as an antioxidant should therefore not rely only on TBARS or fatty acid profile when protein functionality is part of the expected benefit [93,98,99,100]. Polyphenol-rich by-products may delay oxidative deterioration, but their effect on protein oxidation is still less consistently evaluated than their effect on lipid oxidation. Studies that include protein carbonyls, thiol loss, myofibrillar fragmentation, proteolytic enzyme activity and texture measurements provide stronger evidence for technological relevance than studies measuring lipid oxidation alone. This is particularly important when the final product is aged meat, processed meat or fish fillet stored under conditions that favor oxidative changes [9,93,98]. In dairy products, proteolysis is central to cheese ripening and contributes to texture, flavor and bioactive peptide formation. By-product feeding may influence cheese indirectly through milk composition, fatty acid profile, antioxidant capacity and microbial ecology, but direct evidence linking dietary by-products with ripening proteolysis and peptide release remains limited. When dairy products are the final target, studies should evaluate raw milk composition together with cheese-making performance, ripening profile, volatile compounds and sensory traits [21,22,48]. Protein-derived bioactive peptides are a promising but still underdeveloped link between by-product feeding and functional animal-derived foods. Changes in milk or meat protein composition, oxidative status, or processing conditions could affect peptide release during fermentation, ripening, or digestion. Without peptidomic or targeted peptide analyses, however, this remains a hypothesis. Future studies should test this pathway directly before using peptide-mediated functionality as part of the interpretation [9,79,101].

### 7.6. Volatile Compounds and Aroma-Related Pathways

Volatile compounds are final products of several biochemical pathways, including lipid oxidation, amino acid degradation, carbohydrate metabolism, microbial fermentation and Maillard reactions. They are relevant for meat, cheese, eggs and fish because they influence aroma, flavor and consumer acceptance. By-products may modify volatile profiles through changes in fatty acid composition, antioxidant protection, microbial activity or the transfer of aromatic compounds and precursors [9,48]. Lipid-derived volatiles are particularly sensitive to dietary manipulation. Increasing unsaturated fatty acids can improve nutritional value but may also increase the formation of aldehydes, ketones and alcohols during storage or cooking if oxidation is not controlled. Polyphenol- or carotenoid-rich by-products may reduce some oxidation-derived volatiles, whereas citrus residues, olive leaves or aromatic matrices may introduce terpenes or related compounds that influence product aroma. The effect can be favorable or unfavorable depending on dose, product matrix and consumer perception [9,48,97]. In dairy systems, olive leaves and other aromatic by-products may affect milk or cheese volatiles through both direct and indirect mechanisms. Dietary olive leaves in dairy goats have been associated with changes in milk phenolic compounds, antioxidant potential and lipolytic volatile profile, showing that feed bioactives can influence aroma-related pathways as well as nutritional traits [48]. Even so, instrumental VOC changes should not be automatically interpreted as positive. Their sensory relevance depends on concentration, matrix and consumer perception. For meat and fish, volatile compounds are closely linked to oxidation and shelf-life. Lower lipid oxidation may reduce rancidity-associated volatiles, but the effect depends on storage conditions, packaging, cooking and processing. Therefore, by-product feeding studies should interpret VOCs together with fatty acids, oxidation markers and sensory data [46]. These endpoints describe different parts of the same quality pathway and should not be separated when the objective is to support a functional or technological claim [9,79,88].

### 7.7. Integrating Nutritional and Biochemical Pathways with Product-Level Evidence

The pathways discussed in this section are interconnected. Oxidative stress, inflammatory signaling, lipid metabolism, protein oxidation, proteolysis, and volatile formation interact through redox balance, microbial metabolites, fatty acid availability, host gene expression, and post-harvest product chemistry. This explains why the same by-product may improve one trait while having neutral or variable effects on another [9]. Mechanistic studies should therefore avoid relying on a single biomarker. Higher antioxidant enzyme activity is more informative when associated with lower product oxidation. Lipid-metabolism gene expression becomes more useful when accompanied by a modified fatty acid profile. Inflammatory markers are more relevant when linked with health, performance or product traits. Volatile changes need to be interpreted alongside fatty acids, oxidation markers and sensory data. This integrated reading helps distinguish plausible mechanisms from demonstrated product-level effects [79,95,102]. The most convincing studies will combine by-product characterization, microbiome or metabolite analysis, host molecular endpoints and product-level measurements. This is particularly important for a molecularly oriented review, because it keeps the interpretation anchored in chemical entities, metabolic pathways and measurable food traits rather than in broad claims of natural antioxidant activity or sustainability. The main microbiome-mediated and nutritional and biochemical pathways linking by-product feeding with animal-derived food quality are summarized in Table 3 and conceptually integrated in Figure 3 [88,103].

## 8. Omics-Based Traceability and Analytical Platforms

### 8.1. Why Omics Are Needed in By-Product-Based Functional Foods

The development of functional animal-derived foods from agro-industrial by-products requires analytical tools able to follow nutrients and bioactive compounds across feed, animal and product compartments. Conventional measurements such as proximate composition, total phenolic content, antioxidant capacity, fatty acid profile and TBARS remain useful, but they often cannot explain how a by-product changes the final product. In many cases, the key question is not only whether milk, meat, eggs or fish changed, but which substances changed, where they came from, and whether they reflect direct transfer, microbial biotransformation or indirect host-mediated modulation [105,106]. Foodomics provides a useful framework for this purpose because it combines metabolomics, lipidomics, proteomics, volatilomics, genomics and other high-throughput tools to characterize foods at the molecular level. In this review, foodomics is not used as a generic technological label, but as a way to connect feed chemistry, microbiome activity, host response and product composition. This is relevant for by-products because their effects are often mediated by low-abundance metabolites, lipid species, volatile compounds, or peptide profiles that are not captured by routine assays [107,108,109]. The main risk is to generate descriptive fingerprints without biological interpretation. Untargeted omics can identify discriminant components and generate hypotheses, but functional meaning requires a link with experimental design, dose, diet composition, animal response and product-quality endpoints. A metabolite that separates two milk or meat samples is informative only when it can be connected to the by-product, microbial metabolism, host pathways or technological quality. Omics should therefore strengthen the feed-to-food interpretation by connecting statistical discrimination with biologically meaningful changes and measurable product-level pathways [69,106,110].

### 8.2. Targeted and Untargeted Metabolomics

Metabolomics is one of the most useful platforms for tracing the fate of by-product-derived bioactives. It can be applied to feed ingredients, rumen fluid, intestinal digesta, plasma, urine, milk, cheese, meat, eggs and fish tissues, allowing both native compounds and microbial or host-derived metabolites to be detected. This is particularly important for polyphenols, because the nutrient or bioactive components found in the final product are often conjugated or transformed forms rather than the native compounds present in the original by-product [11,106]. Targeted metabolomics is essential when the aim is to confirm transfer of known compounds. Hydroxytyrosol, tyrosol and their sulphate or glucuronide metabolites in dairy products, equol derived from isoflavones, urolithins derived from ellagitannins, and low-molecular-weight phenolic acids derived from flavonoids or fibre-bound phenolics require sensitive and selective methods. LC-MS/MS and UHPLC-MS/MS are particularly suitable because these metabolites may occur at low concentrations and can be missed by non-specific antioxidant assays or by methods targeting only the native dietary compound [21,22,72]. Untargeted metabolomics has a complementary role. It can reveal unexpected metabolites and broader metabolic shifts in biological fluids and products, especially when by-product feeding affects microbial metabolism or host pathways in ways that are not predictable from the feed composition alone. However, unknown features should be interpreted cautiously. Putative annotations need confirmation through MS/MS spectra, authentic standards, or appropriate confidence levels, especially when the result is used to support a functional or traceability claim [111,112]. Metabolomics is strongest when applied across more than one compartment. Measuring the by-product, rumen or intestinal environment, plasma and final product can show whether a compound is present in the feed, transformed by microorganisms, absorbed by the host and finally detected in milk, meat, eggs or fish. This multi-compartment design is demanding, but it provides a stronger basis for distinguishing direct enrichment from metabolite-mediated transfer or indirect quality modulation [69,110].

### 8.3. Lipidomics and Fatty Acid-Centered Approaches

Fatty acid analysis is already common in by-product feeding studies, especially when the objective is to improve milk fat, meat lipids, egg yolk or fish fillets. Lipidomics can extend this information by identifying lipid classes and intermediary products, including triacylglycerols, phospholipids, sphingolipids, cholesterol esters and oxidized lipids. This is useful because the biological and technological behaviour of a product depends not only on the amount of saturated, monounsaturated or polyunsaturated fatty acids, but also on how these fatty acids are distributed across lipid fractions [113,114]. In ruminants, lipidomics can help clarify whether by-products influence product lipids through dietary lipid supply, ruminal biohydrogenation or host lipid metabolism. Grape, olive, pomegranate and tomato by-products may alter milk fatty acid profile, but the mechanism may differ among matrices. Changes in vaccenic, rumenic or linolenic acids may reflect microbial effects in the rumen, residual lipids in the by-product, or altered mammary lipid synthesis [6,13,16]. Lipidomics can support this interpretation when combined with rumen fermentation data, biohydrogenation intermediates, and product-level fatty acid profile. In meat and fish, lipidomics is particularly relevant because oxidative stability and nutritional quality are closely linked. Products richer in unsaturated lipids may have a better nutritional profile but can also be more prone to oxidation during storage, processing, or cooking. Lipidomics can identify oxidized lipid species and lipid classes that are not captured by total fatty acid analysis alone. This is especially useful in fish and seafood, where long-chain polyunsaturated fatty acids are abundant, and oxidation strongly affects shelf-life and sensory quality [99,114]. For eggs, lipidomics could improve the interpretation of yolk enrichment. Carotenoid-rich or lipid-rich by-products may modify yolk pigmentation, fatty acid composition and oxidative stability, but the functional relevance depends on the stability and distribution of lipophilic compounds in the yolk matrix. Combining carotenoid analysis, fatty acid profile, lipid oxidation and lipid class information would provide a stronger basis for interpreting egg enrichment studies than using lipidomics alone [25,26,42].

### 8.4. Volatilomics and Aroma Profiling

Volatile compounds are key markers of product quality because they contribute to aroma, flavor, freshness, and oxidation-related off-flavors. Volatilomics can therefore help explain how by-product feeding affects sensory and technological traits of milk, cheese, meat, eggs and fish. PTR-ToF-MS, GC-MS and related approaches can detect volatile fingerprints associated with lipid oxidation, microbial fermentation, amino acid degradation, terpenes and processing-derived reactions [115,116]. In by-product feeding studies, volatilomics is useful for two main reasons. First, it can identify oxidation-related volatiles, such as aldehydes, ketones and alcohols, which are relevant for meat, fish and dairy shelf-life. Second, it can detect aromatic compounds or precursors derived from plant residues, especially when citrus, olive leaves, grape pomace or other aromatic matrices are used. These changes may improve or impair product acceptability depending on concentration, matrix and consumer perception [9,117]. Volatile profiling should not be interpreted alone. A change in VOC pattern may reflect lipid oxidation, microbial fermentation, altered fatty acid profile, feed-derived aromatic compounds or processing conditions. For this reason, volatilomics should be linked with fatty acid profile, lipid and protein oxidation, microbial data, sensory evaluation and storage conditions [46]. This is particularly important for dry-aged meat, ripened cheese and fish fillets, where volatile compounds evolve during post-harvest processing rather than only during animal feeding [79,115].

### 8.5. Proteomics, Peptidomics and Protein Oxidation

Proteomics and peptidomics are less commonly used than metabolomics or fatty acid analysis in by-product feeding studies, but they are relevant for meat tenderness, water-holding capacity, cheese ripening, peptide formation and protein oxidation. In meat, proteomics can identify changes in myofibrillar, sarcoplasmic and mitochondrial proteins associated with tenderness, color stability, water retention and post-mortem metabolism [100,118]. In dairy products, proteomics and peptidomics can describe casein degradation, whey protein changes, ripening dynamics and the formation of bioactive peptides [101]. By-product feeding may influence protein quality indirectly through oxidative status, inflammation, nutrient partitioning or milk composition. Antioxidant-rich residues may reduce protein oxidation in meat, with possible consequences for tenderness, water-holding capacity and processing behavior. In cheese, changes in milk composition or antioxidant environment may influence ripening proteolysis and peptide formation. These mechanisms are plausible, but most feeding trials still focus more on lipids than proteins [79,93,100]. Peptidomics may become useful when the objective is to discuss functional dairy or meat products. Fermentation, ripening and digestion can release peptides with antihypertensive, antioxidant, antimicrobial or immunomodulatory potential, but it is still unclear whether by-product feeding can reproducibly influence this peptide profile. Studies testing this hypothesis should include product processing, peptide identification, bioactivity assays and, where appropriate, simulated digestion. Without this information, claims about peptide-mediated functionality remain speculative [100,101]. Protein oxidation should also be integrated with product quality. Carbonyl formation, thiol loss and oxidative modifications can affect muscle structure, enzyme activity and proteolysis, thereby influencing texture and technological properties. If a by-product is proposed as an antioxidant strategy for meat or fish, measuring only lipid oxidation gives an incomplete picture. Protein oxidation markers, proteomic changes and texture data should be included to understand whether the feeding strategy improves the stability of the whole product matrix [79,93,99,100].

### 8.6. Multi-Omics Integration Across Feed, Microbiome, Host and Product

The strongest evidence for by-product-driven functional animal-derived foods will come from designs that connect the feed matrix with microbial activity, host metabolism and the final product. A complete study could include by-product metabolomics, rumen or gut microbiome profiling, rumen or intestinal metabolomics, plasma metabolomics, tissue or milk transcriptomics, product lipidomics and product metabolomics. Such a design is demanding, but it is the most appropriate way to distinguish direct transfer from microbial transformation and indirect host-mediated modulation [69,110,119]. The dairy cow provides a useful model. Multi-omics work has shown that rumen microbiome, rumen metabolome and host metabolome together contribute to individualized dairy cow performance, indicating that microbial and host metabolic layers are closely connected [69]. For by-products, this means that the same feed matrix may produce different product outcomes depending on the animal’s microbial and metabolic phenotype. Integrated analyses are therefore more informative than isolated measurements of feed composition or milk quality. Multi-omics can also help identify biomarkers of feeding strategy. Candidate markers may include polyphenol metabolites, lipid species, volatile compounds, carotenoids, peptides, microbial metabolites or combinations of these compounds and their intermediates. Such markers could support product traceability, authentication of circular feeding systems and verification of functional enrichment. However, biomarker discovery requires validation in independent populations and under real production conditions. Without validation, omics markers remain exploratory only and should not be used as definitive proof of origin or functionality [105,110,119]. Integration is not only a statistical problem. Multi-omics data should be interpreted according to biological sequence: by-product composition, processing, microbial transformation, host response and product traits. Correlation networks can identify associations, but they cannot prove causality unless supported by experimental design, time-course sampling, dose-response evidence and mechanistic validation. This is particularly important in by-product studies, where many dietary components change at the same time and the product effect may result from combined changes in fibre, lipid, protein, polyphenols and fermentability [69,110,119].

### 8.7. Practical Limitations and Minimum Analytical Standards

Omics technologies are powerful, but they are not always feasible for routine feeding trials. Cost, sample size, analytical expertise, data processing, and metabolite annotation remain important constraints. Not every study using agro-industrial by-products needs full multi-omics analysis. However, studies that aim to support functional-food claims should include a minimum analytical framework linking by-product characterization with product-level measurements, adapted to the expected mechanism [9,106,110]. For polyphenol-rich by-products, this framework should include characterization of the feed matrix, targeted analysis of selected metabolites in biological fluids or products, and product-level oxidative, sensory or shelf-life endpoints. For carotenoid-rich by-products, carotenoid retention after processing and deposition in the edible product should be measured. For lipid-rich residues, fatty acid profile should be interpreted together with oxidative stability and, where possible, lipid class analysis. For fibre-rich residues, microbiome or fermentation data should be related to product outcomes rather than reported as isolated gut-health endpoints [21,25,29,114]. Analytical transparency is essential, and studies should report sample handling, storage conditions, extraction protocols, quality-control samples, analytical platforms, identification confidence, normalization procedures and statistical criteria. This is particularly important for metabolomics, lipidomics and volatilomics, where pre-analytical variation can strongly influence results. Without adequate reporting, omics data may appear sophisticated but remain difficult to reproduce or compare across studies [106,111,112]. A practical standard can therefore be proposed. Basic feeding studies should report by-product composition, processing conditions, inclusion level and product-quality endpoints. Mechanistic studies should add targeted metabolites, microbial or host markers and time-course or dose-response information. Studies claiming functional enrichment should demonstrate the relevant compound, metabolite or validated quality effect in the final edible product. This hierarchy keeps omics interpretation proportional to the strength of the evidence and avoids turning exploratory fingerprints into unsupported functional claims.

## 9. Circular Valorization, Feed Safety and Regulatory Constraints

### 9.1. Circularity Is Not Automatically Functionality

Agro-industrial by-products are often presented as circular feed resources, but their practical translation into high-value animal-derived foods also depends on safety, logistics, regulatory classification, scalability and reproducible product-level effects. A residue may reduce waste and partially replace conventional ingredients, yet its real value depends on the cost of the product, nutrient recovery, local availability, processing needs, transport distance, safety, animal performance, and final product quality. Circular valorization is therefore a chain-level outcome, not an intrinsic property of the by-product [7,120,121]. This point is especially relevant when processing intensity increases. A moist residue used locally after ensiling has a different circular profile from the same material dried, purified, or transported over long distances. Likewise, a whole by-product used as a feed ingredient and a concentrated extract used as a standardized bioactive source may both originate from residues, but they differ in nutrient-recovery value, energy demand, cost and functional objective [7,57,58]. From a functional-food perspective, circularity becomes meaningful only when it is related to measurable animal gain and product quality outcomes. A feeding strategy can be circular and nutritionally useful without generating a functional animal-derived food, whereas an extract can improve product quality while contributing little to nutrient recycling. The strongest evidence comes from studies that evaluate residue valorization, feed safety, animal performance, product-quality improvement and environmental feasibility within the same production chain [8,9,121].

### 9.2. Life-Cycle Thinking and Substitution Effects

Life-cycle thinking is needed to understand whether waste-to-feed strategies actually reduce environmental burdens. The advantage of using by-products is not simply that a residue is diverted from disposal, but that it may replace conventional feed materials whose production requires land, water, fertilizers, fuel and transport. The environmental benefit therefore depends on substitution ratio, nutritional equivalence, processing energy, transport distance and allocation method [121,122]. To make this interpretation more transparent, Supplementary Table S5 summarizes the main life-cycle-related indicators and expected environmental or economic trade-offs associated with different by-product processing routes, including local ensiling, drying, fermentation, fractionation and extract-based strategies. Food-loss and food-waste-to-feed pathways can be environmentally advantageous, but the benefit is case-specific. Key variables include the original waste-management scenario, drying or stabilization requirements, distance between production and use, feed formulation constraints, and the ingredient being replaced. High-moisture residues are a clear example: local ensiling may preserve circular value, whereas energy-intensive drying can reduce the advantage if it is not offset by storage stability, nutrient recovery, or product-quality benefits [120,121]. Allocation is another critical issue. A by-product may be treated as waste with low environmental burden, as a co-product carrying part of the impact of the primary process, or as an ingredient with a market value. These choices can change the result of a life-cycle assessment. A residue with no local market may be valuable when used in nearby farms, whereas a high-demand co-product may no longer represent a low-impact alternative if its use creates competition with other sectors [121,122]. Environmental indicators should also be read together with product quality. A by-product that slightly reduces environmental impact but worsens shelf-life, sensory traits, or consumer acceptance has limited practical value. Taken together, these aspects indicate that circular valorization should be interpreted together with animal performance and final product quality. The most valuable strategies are those in which by-product use reduces waste or feed competition while also maintaining performance and improving oxidative stability, nutritional value, shelf-life or other measurable product-level traits beyond simple feed substitution [7,9,121]. Therefore, circular valorization should not be evaluated only by the origin of the ingredient, but by the balance between avoided waste, feed substitution, processing intensity, transport distance, safety control, cost and measurable product-quality benefit.

### 9.3. Feed Safety: Contaminants, Spoilage and Carry-Over Risks

Feed safety remains one of the main constraints in by-product use. Residues may carry mycotoxins, pesticide residues, heavy metals, dioxins, spoilage microorganisms, lipid oxidation products or process-related contaminants, depending on crop origin, industrial process, storage conditions and stabilization method. Cereal by-products, oilseed cakes, fruit pomaces, vegetable residues and mixed waste streams deserve particular attention because variability and moisture can complicate quality control [3,123,124]. Mycotoxins are especially relevant because they may contaminate raw materials before processing and persist in the resulting by-products. Drying or ensiling cannot be assumed to eliminate the risk. Some mycotoxins can affect animal health and performance, and selected compounds or metabolites may carry over into animal-derived foods under specific conditions. Mycotoxin monitoring should therefore be part of the minimum safety assessment for by-products intended for feed use [123,124,125]. Pesticide residues and heavy metals require the same caution. Fruit and vegetable residues may reflect pre-harvest treatments, while mineral contaminants may derive from soil, water, industrial equipment or processing environments. This issue becomes more delicate for concentrated extracts, because extraction may enrich beneficial molecules but may also concentrate unwanted residues if selectivity and purification are insufficient [3,58]. Microbial spoilage is a practical risk for high-moisture residues such as fruit pomaces, citrus pulp, tomato pomace and vegetable wastes. These materials can ferment rapidly and develop undesirable microbial populations if they are not stabilized soon after production. Ensiling and fermentation can improve safety when well controlled, but poor fermentation may generate spoilage, biogenic amines, off-odors, nutrient losses or reduced palatability. Processed by-products should therefore be evaluated after stabilization, not only as fresh residues [3,7,18]. Lipid-rich residues add another risk. Grape seeds, tomato seeds, oilseed cakes and other matrices containing unsaturated lipids may improve fatty acid profile, but they may also introduce oxidized lipids if processing and storage are inadequate. Peroxide value, secondary oxidation products and storage conditions should be considered when these residues are used to improve the nutritional quality of meat, eggs, milk or fish products [3,9,79].

### 9.4. Standardization and Batch-to-Batch Variability

Standardization is one of the main barriers to routine use of by-products in animal feeding. Composition can vary between years, factories, cultivars, processing lines, and batches from the same plant. This variability affects dry matter, protein, fibre, lipid content, phenolic profile, carotenoid retention, fermentability, anti-nutritional factors and contaminants. Without adequate characterization, it is difficult to define inclusion levels, compare studies or reproduce product-quality effects [2,7,19]. Phenolic-rich residues illustrate the problem well. Grape pomace may differ in seed-to-skin ratio, tannin concentration, anthocyanin profile and residual oil. Olive by-products vary with cultivar, extraction system, pulp/pit/skin proportion and storage conditions. These differences can change both diet formulation value and biological activity. Trials should therefore report the actual composition of the material used, not only its commercial name or industrial origin [3,19,20]. Standardization also affects dose-response interpretation. Many studies test one or two inclusion levels without quantifying the active molecular dose. Reporting inclusion as percentage of diet dry matter is useful, but not enough when the expected mechanism depends on phenolics, tannins, carotenoids, tocopherols or specific fatty acids. When possible, the active dose should be expressed as intake of relevant compounds per animal per day or per kg of dry matter intake [9,16]. Quality control should therefore combine nutritional and molecular descriptors. For practical feeding, dry matter, protein, fibre, fat, ash and energy value remain essential. For functional-food development, additional markers are needed, including individual phenolics, tannin fractions, carotenoids, tocopherols, fatty acids, antioxidant capacity, fermentation quality and safety parameters. Full omics analysis is not required for every commercial batch, but functional claims need a more detailed specification than conventional feed use [2,3,110].

### 9.5. Scalability, Logistics and Economic Feasibility

Scalability often determines whether a promising by-product remains an experimental ingredient or becomes a practical feeding solution. Many residues are produced seasonally and locally, while livestock systems require stable supply, predictable composition and reliable storage. A material that performs well in a trial may be difficult to use commercially if production is seasonal, moisture is high, transport is expensive, or spoilage occurs rapidly [7,120,121]. Local integration between food-processing plants and farms is often the most realistic model. Mediterranean vegetable residues such as artichoke bracts are a useful example: their use as silage in finishing beef diets links territorial availability, stabilization strategy and meat-quality evaluation within the same production chain, especially when the final product is assessed after a value-adding process such as dry aging [56]. High-moisture and low-bulk-density residues are generally better suited to regional use, ensiling or co-ensiling than to long-distance trade. Economic feasibility should include more than ingredient price. Transport, stabilization, storage losses, analytical controls, formulation constraints, effects on intake or performance, and the market value of improved product quality all influence the real value of the strategy. A by-product that improves oxidative stability or shelf-life may generate economic value beyond feed-cost reduction. Conversely, a low-cost residue becomes less attractive if it reduces palatability, intake or product yield [7,9,120]. Industrial involvement can improve scalability through standardized supply, quality control and traceability. Feed companies and food processors may also support fermented ingredients, processed fractions or standardized extracts. However, once a residue becomes a high-value ingredient, its availability, price and environmental allocation may change. This dynamic should be considered when moving from proof-of-concept to on-farm experimental feeding to commercial application stage [58,121,122].

### 9.6. Regulatory Aspects, Functional-Food Claims and Practical Selection Criteria

The regulatory classification of by-products, processed ingredients and extracts is not always straightforward. A dried whole by-product, an ensiled residue, a fermented ingredient and a purified bioactive extract may fall into different categories depending on production process, intended use, inclusion level and country. This is particularly relevant when the ingredient is no longer used only as a feed material, but as a standardized bioactive source intended to modify the final animal-derived food [58,123,124]. Feed materials must comply with safety, traceability and risk-control requirements for animals, consumers and the environment. The circular origin of a material does not reduce the need for documented controls; in some cases, it increases it because residues are variable and may carry contaminants or spoilage risks. Mycotoxins, heavy metals, dioxins, PCBs, pesticide residues and microbial hazards require attention according to the matrix and production chain [123,124,125]. Functional-food claims require an additional level of caution. Improving fatty acid profile, antioxidant capacity or shelf-life does not automatically justify health-related claims for consumers. A product may be nutritionally improved or technologically more stable without meeting the scientific or regulatory requirements for a health claim. Manuscripts should therefore distinguish product-quality improvement, nutritional enrichment, technological functionality and consumer health claims [9,26]. Consumer communication also matters. Circular feeding may be perceived positively when associated with sustainability, waste reduction and local valorization, but it may raise concerns if by-products are misused with unmanaged waste. Communication should emphasize that these materials are controlled feed ingredients, not uncontrolled residues entering the food chain. At the same time, claims of naturalness, sustainability or health benefit should remain proportional to analytical, safety and product-quality evidence [9,120,121]. A practical selection pathway should start from legal usability, feed safety, local availability and nutritional role. Once these requirements are satisfied, the matrix can be evaluated for the nutrient or bioactive substance relevant to the expected product outcome, such as polyphenols, carotenoids, fermentable fibres, lipid fractions or residual proteins. Feeding trials should then use realistic inclusion levels, appropriate controls and product-level endpoints, with microbial, metabolomic, lipidomic or oxidative markers included when they are needed to support the proposed mechanism [3,7,124].

## 10. Knowledge Gaps and Future Research Priorities

The literature on agro-industrial by-products in animal feeding is now broad. The main gap is no longer the lack of candidate ingredients, but the limited number of studies that follow the complete pathway from by-product characterization to animal response, microbial or host-mediated transformation, and final product quality [7,110]. Future research should move from asking whether a by-product “works” to defining under which conditions it works, through which mechanism, and with which measurable effect on the final animal-derived food. The first step is better characterization of the material actually selected for inclusion in the diet. Proximate composition, fibre fractions and inclusion level are necessary, but they are not sufficient when product-quality changes are attributed to specific nutritional and biochemical pathways. Polyphenol-rich residues require the characterization of individual phenolic acids, flavonoids and tannin fractions; carotenoid-rich matrices require lycopene, β-carotene and other pigments after processing; lipid-rich residues require fatty acid profile and oxidation status; fibre-rich by-products require information on fermentability and cell-wall-bound polyphenols [2,19,26,28]. The final processed ingredient should be analysed, not only the raw residue or values taken from the literature [3,20,31]. The second priority is dose-response evidence. Many studies test a single inclusion level, or at most two levels, without clarifying whether the aim is nutritional replacement, bioactive supplementation or both. This makes it difficult to identify the optimum inclusion rate and to distinguish beneficial, neutral and negative effects. Dose-response information is especially important for tannin-rich, lipid-rich, fibre-rich and aromatic residues, where higher inclusion may reduce palatability, digestibility, intake or sensory acceptability [13,14,70]. The most relevant dose is not simply the highest safe level, but the level that maintains performance while producing a desirable product quality effect [6,23,25,42]. The third priority is integration. Many studies measure animal performance and product traits, whereas others focus on rumen or gut microbiota. Fewer studies connect by-product composition, microbial transformation, host metabolism, and final food quality within the same design. This is particularly important for polyphenol-rich by-products, because native compounds may be degraded, converted into phenolic acids, transformed into metabolites such as equol or urolithins, or conjugated by the host before reaching milk, cheese, meat or eggs [11,22,27,72]. The fourth priority is terminology. Functional claims should be based on the edible product, not on the feed matrix alone. “Enrichment” should be reserved for cases in which a defined nutrient, bioactive substance, or their intermediate metabolite increases in milk, cheese, meat, eggs, or fish. “Quality modulation” is more appropriate when the diet improves fatty acid profile, oxidative stability, color, volatile compounds, technological traits, shelf-life or sensory traits without proving direct deposition of a specific compound [9,26]. Finally, feed safety and sustainability should be built into experimental designs. Mycotoxins, pesticide residues, heavy metals, spoilage microorganisms, oxidized lipids and solvent residues may limit by-product use, especially when processing concentrates selected fractions. Likewise, a strategy that improves bioactive recovery may lose practical value if it requires high energy input, long transport, expensive purification or complex waste management [3,58,123,124]. The most useful future studies will combine realistic feeding conditions, practical applicability, product-quality validation, safety controls and simplified sustainability or scalability indicators.

## 11. An Integrated Framework for Future Studies and Applications

### 11.1. From Opportunistic Use to Mechanism-Driven Design

Future work should move from opportunistic inclusion of available residues to mechanism-driven experimental design. In many studies, a by-product is selected because it is local, inexpensive, or nutritionally usable, and functional effects are explored afterwards. This approach is useful for screening, but it is not enough for developing credible functional animal-derived foods. Stronger studies should begin with a clear hypothesis: which by-product is being used, which nutrients or matrix features are expected to be bioactive, which microbial or host pathways are involved, and which product-level outcome should change [9,110]. The proposed framework follows five connected steps: matrix selection and characterization; processing, safety and stability validation; animal trial design according to dose and mechanism; measurement of biological intermediates; and final assessment of product quality and circular value. These steps belong to the same feed–microbiome–host–food continuum. This structure is consistent with the purpose of scoping reviews, which is not only to summarize evidence, but also to identify gaps and guide future research priorities [37,126].

### 11.2. Step 1: Matrix Selection and Molecular Characterization

The first step is the selection of a matrix that is both practically usable and nutritionally relevant. Local availability, seasonality, price, and compatibility with the target species are necessary, but they should be considered together with the expected functional fraction. Examples include polyphenols in grape and olive residues, flavanones and pectin in citrus pulp, carotenoids in tomato pomace, fibre-bound phenolics in cereal brans, and residual lipids or proteins in oilseed cakes and brewer’s spent grain [2,19,20,31]. The actual material included in the diet should be analysed. Cultivar, industrial process, recovered fraction, drying method, storage time and batch can change the matrix substantially. For functional-food purposes, proximate composition should be complemented with targeted molecular descriptors, including phenolic profile, tannin fractions, carotenoids, tocopherols, lipid profile, oxidation status, fibre structure, fermentability and relevant contaminants. Without this information, active dose, study comparison and mechanistic interpretation remain weak [3,28].

### 11.3. Step 2: Processing, Safety and Stability Validation

The second step is validation of the processed ingredient before the animal trial. Drying, ensiling, fermentation, enzymatic treatment and extraction can preserve bioactives or increase bioaccessibility, but they may also reduce biological relevance by degrading sensitive compounds, modifying the original matrix or increasing processing intensity. The stabilized material should therefore be analysed after processing, because its nutritional profile may differ substantially from that of the fresh residue [18,57,58]. Safety and stability belong to the same step. Moisture, microbial quality, mycotoxins, pesticide residues, heavy metals, lipid oxidation and, when relevant, solvent residues or fermentation products should be checked according to the matrix and processing method. This is particularly important for high-moisture residues, cereal by-products, oilseed cakes, fruit pomaces and concentrated extracts. A by-product cannot be considered functional or circular if it introduces uncontrolled risks into the feed–animal–food chain [3,123,124].

### 11.4. Step 3: Animal Trial Design and Dose Definition

The third step is an animal trial coherent with the expected mechanism. If the objective is nutritional replacement, the diet should maintain comparable energy, protein, fibre and lipid supply. If the objective is bioactive supplementation, the active component should be quantified and reported. If the objective is functional product development, the trial should include endpoints in the final food, not only animal performance. Replacement, supplementation and functional enrichment are different experimental aims and should not be merged in the interpretation [7,9,16]. Dose-response designs are preferable whenever possible. A single inclusion level can show feasibility, but it rarely identifies the best balance between performance, biological response and product quality. This is relevant for tannin-rich residues, lipid-rich by-products, aromatic matrices and concentrated extracts, where higher doses may reduce intake, digestibility, palatability or sensory acceptability. Dose should be expressed not only as a percentage of diet on a dry matter basis, but also as intake of relevant active compounds when the mechanism depends on polyphenols, carotenoids, tannins, tocopherols or specific fatty acids [13,14,70].

### 11.5. Step 4: Biological Intermediates and Product-Level Validation

The fourth step connects biological intermediates with the final food. In ruminants, relevant intermediates may include rumen fermentation, microbiota, biohydrogenation intermediates, methane-related variables, plasma metabolites, and mammary or tissue lipid metabolism. In monogastrics, gut microbiota, short-chain fatty acids, intestinal barrier markers and systemic oxidative or inflammatory indicators may be more informative. These measurements are needed because many by-products act through microbial transformation or indirect host-mediated pathways rather than through direct transfer of native compounds [13,69]. Targeted analyses should match the expected mechanism and identification of end-product and its biological effect or functional attributes stated as examples below [46]. Olive-derived polyphenols require measurement of hydroxytyrosol, tyrosol and conjugated metabolites. Tomato-derived matrices require carotenoid retention and deposition. Tannin-rich residues require rumen fermentation, nitrogen metabolism and biohydrogenation data. Fibre-rich residues require fermentation and microbial metabolite information. Untargeted omics can support discovery, but targeted validation is needed when enrichment or specific metabolic transfer is claimed [21,22,25]. Product-level analysis should then reflect the expected effect. Milk and dairy products require composition, fatty acid profile, phenolic compounds, antioxidant capacity, functional properties, volatile compounds and ripening traits. Meat and fish require fatty acids, lipid and protein oxidation, color stability, volatile profile, shelf-life and sensory traits. Eggs require yolk carotenoids, fatty acids, lipid oxidation, pigment stability and sensory acceptability. Without product-level validation, the study cannot support a functional-food interpretation [9,26,79]. The final terminology should follow the evidence. Enrichment is appropriate when a defined compound or metabolite increases in the edible product. Quality modulation is more accurate when the by-product improves fatty acid profile, oxidative stability, color, volatile profile, or shelf-life without demonstrating deposition of a specific compound. If evidence is limited to feed composition, in vitro antioxidant capacity or in vivo animal performance, the claim should remain mechanistic or preliminary [21,23,25].

### 11.6. Step 5: Circularity, Feasibility and Decision Pathway

**Figure 4:**
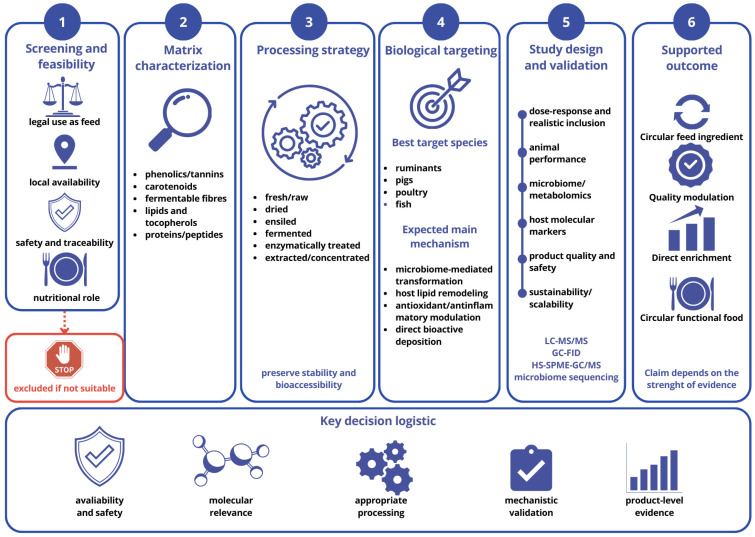
Proposed decision framework for precision circular feeding using agro-industrial by-products.

The final step is the integration of circularity and industrial feasibility. Local availability, transport distance, processing energy, storage stability, substitution of conventional feedstuffs, safety controls and scalability should be considered before a by-product is proposed for routine use. A biologically effective ingredient may have limited practical value if it is available only seasonally, requires expensive drying, varies strongly between batches or cannot be standardized under commercial conditions [7,121,122]. The most robust applications are those in which circular valorization, biological actions, and product functionality support each other. A whole by-product may be preferable when the aim is nutrient recovery, local reuse and partial replacement of conventional feed ingredients. A concentrated fraction may be preferable when the aim is standardized bioactive supplementation or mechanistic testing. In both cases, the claim should depend on the evidence: circular feed ingredient, quality modulation, direct enrichment or circular functional food. The decision pathway proposed in Figure 4 integrates these criteria and moves from residue availability to safety, nutritional relevance, processing, biological validation and final product-quality evidence [57,58]. The same by-product should not be considered universally useful across species and products. Carotenoid-rich matrices may be particularly suitable for eggs because yolk deposition is relatively direct. Polyphenol-rich residues may be more useful for ruminant milk or meat when they influence rumen biohydrogenation, oxidative stability or metabolite transfer. Fibre-rich residues may be more suitable for ruminants, or for monogastrics when processing improves fermentability and digestibility. Lipid-rich residues should be targeted to products where fatty acid improvement can be achieved without compromising oxidative stability [6,25,42]. Ultimately, the strongest studies will combine circular logic with product-level precision. They should clarify whether the by-product is used for nutrient recovery, targeted enrichment, metabolite-mediated transfer, preservation of product quality beyond the farm gate, or a combination of these aims. A useful by-product should be locally available, safe, nutritionally relevant, processable at reasonable cost, and able to generate a reproducible product-level effect. When these conditions are met, agro-industrial by-products can move beyond the role of alternative feed ingredients and become credible tools for producing functional animal-derived foods within circular food systems [7,9,121].

## 12. Conclusions

Agro-industrial by-products represent more than low-cost feed substitutes or generic circular economy tools. Their scientific value lies in the possibility of linking nutrient recovery with targeted metabolic modulation and measurable improvements in animal-derived foods. However, biological functionality cannot be inferred from botanical origin, industrial source or gross chemical composition alone. The evidence reviewed across the feed–microbiome–host–food continuum shows that the response to by-product feeding is strongly matrix-, dose-, species- and process-dependent. Therefore, by-product valorization should be interpreted through differentiated feeding scenarios, active-compound availability, species-specific digestive physiology and product-level endpoints rather than through generic assumptions of functional value.

A key conclusion is that ruminant and monogastric species require different interpretative and control schemes. In ruminants, including dairy cows, sheep and goats, extensive pre-absorptive microbial transformation occurs in the rumen. Tannin–protein interactions, microbial hydrolysis, aromatic ring cleavage, nitrogen metabolism and lipid biohydrogenation can strongly influence the final response in milk, cheese and meat. Product modulation in ruminants is therefore often mediated through altered ruminal fermentation, changes in vaccenic and rumenic acid intermediates, improved oxidative stability or the transfer of microbial and host-derived metabolites. In monogastric species, including poultry, pigs and several aquaculture species, dietary bioactives partly bypass ruminal degradation. Some lipophilic compounds may be absorbed more directly and deposited in edible tissues or egg yolk, whereas poorly absorbed tannins, flavonoid glycosides and fibre-bound polyphenols may reach the hindgut and be converted into short-chain fatty acids and low-molecular-weight phenolic metabolites. These differences indicate that ruminant strategies should prioritize ruminal biohydrogenation, nitrogen efficiency and metabolite transfer, whereas monogastric strategies should focus on bioaccessibility, cell-wall disruption, direct deposition, hindgut fermentation and control of anti-nutritional or oxidative constraints.

Among the main by-product categories, grape-derived residues appear particularly relevant for ruminant lipid and oxidative modulation and may also have selected applications in aquaculture. Reported inclusion levels include approximately 10% of diet dry matter in lambs, up to 6% of diet dry matter for dried grape pomace in dairy goats, 5–15% of diet dry matter for ensiled grape pomace in dairy goats, and species-dependent levels in aquafeeds, such as 0.4% in European sea bass and 5–10% in common carp. These values should be interpreted as evaluated ranges under specific experimental conditions, not as universal recommendations. Overall, grape residues may support changes in ruminal biohydrogenation, fatty acid profile, volatile organic compounds and oxidative stability, but practical use requires control of tannin reactivity, palatability, fibre digestibility, processing method and batch-to-batch variability. Olive-derived by-products, including olive pomace, olive leaves and olive mill wastewater-derived fractions, are most strongly supported in dairy small ruminants and are also emerging in circular feeding strategies for monogastric species. Their relevance is associated with hydroxytyrosol, tyrosol, oleuropein derivatives and related phenolic metabolites. Olive matrices provide one of the clearest examples of metabolite-mediated transfer, because hydroxytyrosol, tyrosol and conjugated metabolites can be detected in milk and cheese when targeted analytical methods are used. Nevertheless, whole residues and standardized polyphenol-rich extracts should not be interpreted as equivalent interventions. They differ in circular value, molecular precision, processing intensity, regulatory classification, cost and potential risk of concentrating contaminants. Tomato processing by-products provide one of the clearest examples of direct feed-to-food carotenoid enrichment, particularly in poultry. Tomato powder at 5–10 g/kg diet in laying hens has been associated with increased yolk carotenoids, retinol-related traits, pigmentation and reduced lipid peroxidation. In meat-producing ruminants, tomato pomace is more appropriately interpreted as a circular concentrate replacer and antioxidant-related matrix, with potential effects on oxidative stability and meat quality rather than as a simple direct carotenoid-enrichment strategy. Citrus pulp represents a different scenario, acting mainly as a fermentable fibre and energy source in dairy ruminants. Reported inclusion levels of approximately 9–18% of diet dry matter may support milk antioxidant-related traits, including total polyphenols, flavonoids and ferric-reducing antioxidant power, but these effects still require product-level validation and careful diet formulation. Other by-product categories should be interpreted according to their dominant matrix function. Cereal brans and brewer’s spent grain are mainly structural carbohydrate and protein sources, and their functional potential depends on the release of cell-wall-bound compounds, such as ferulic acid, through processing, fermentation or enzymatic upgrading. Oilseed cakes and seed-rich residues may contribute residual lipids and protein but require control of oxidative stability and digestibility. Ensiled vegetable residues, such as artichoke bracts or mixed carrot, potato and tomato residues, are best classified as circular roughage or concentrate replacers that may support animal performance and product quality during post-harvest processes, such as dry aging, rather than as direct bioactive-transfer agents unless specific dietary metabolites are verified in edible tissues.

Overall, the strongest category-specific conclusions support grape-derived residues for ruminant lipid and oxidative modulation, olive-derived polyphenols for metabolite enrichment in dairy products, tomato-derived carotenoids for direct yolk deposition in eggs, and citrus pulp as a fermentable matrix with potential antioxidant-related effects in dairy cows. For cereal, brewery, oilseed and vegetable residues, current evidence more strongly supports circular feeding value and indirect quality modulation than direct functional transfer. Future research should report active-compound dose, processing history, safety parameters, microbiome and metabolomic responses, product-level validation and simplified life-cycle or scalability indicators. Functional claims should be classified according to three distinct levels of evidence: direct compound enrichment, metabolite-mediated transfer or indirect quality modulation. Only through category-specific, species-tailored and mechanism-driven validation can agro-industrial by-products be translated into scientifically credible, high-value animal-derived foods within precision circular feeding systems.

## Figures and Tables

**Table 1:** Main agro-industrial by-products, dominant bioactive fractions and expected functional relevance for animal-derived foods.

By-Product or Residue	Main Molecular Fractions	Main Functional Rationale	Most Relevant Animal-Derived Products	Critical limitations	References
Grape pomace, grape seeds and winery residues	Condensed tannins, anthocyanins, flavanols, phenolic acids, stilbenes, seed lipids	Modulation of rumen biohydrogenation, oxidative stability, fatty acid profile and volatile formation	Milk, cheese, meat, fish fillets, eggs	High batch variability; tannin dose; palatability; fibre digestibility	[2,13,19]
Olive pomace, olive cake, olive leaves and olive mill wastewater	Hydroxytyrosol, tyrosol, oleuropein derivatives, verbascoside, secoiridoids, oleic acid	Antioxidant modulation, lipid stability, phenolic metabolite transfer, milk and meat fatty acid modulation	Milk, cheese, meat	Variable phenolic profile; bitterness; lipid oxidation; regulatory differences between whole residues and extracts	[20,21,22]
Citrus pulp and citrus peel	Pectin, soluble carbohydrates, hesperidin, naringin, limonene and related terpenes	Fermentation modulation, antioxidant-related milk traits, possible volatile effects	Milk, dairy products, meat, monogastric products	Moisture; palatability; terpene transfer; need for stabilization	[3,4,23]
Tomato pomace and tomato-processing residues	Lycopene, β-carotene, tocopherols, seed oil, fibre, phenolic compounds	Carotenoid deposition, yolk pigmentation, antioxidant protection, lipid-quality modulation	Eggs, poultry meat, fish fillets, milk and meat	Lycopene bioaccessibility; drying losses; oxidative degradation during storage	[24,25,26]
Pomegranate peel and fruit residues	Ellagitannins, ellagic acid, anthocyanins, phenolic acids	Microbial conversion to urolithins, antioxidant and anti-inflammatory modulation, lipid oxidation control	Milk, meat, dairy products	Limited direct product-level evidence; metabotype-dependent conversion; high tannin reactivity	[9,11,27]
Cereal brans	Arabinoxylans, ferulic acid, p-coumaric acid, fibre, minerals, residual proteins	Fibre fermentation, release of bound phenolics, gut and rumen modulation	Milk, meat, eggs, monogastric products	Bound phenolics; variable fermentability; need for enzymatic or microbial upgrading	[28,29,30]
Brewer’s spent grain	Arabinoxylans, cellulose, lignin, residual proteins, bound phenolic acids	Protein and fibre recovery, microbial fermentation, possible phenolic release after processing	Ruminant products, pork, poultry, aquaculture products	High moisture; spoilage risk; low digestibility if untreated; batch variability	[7,31,32]
Oilseed cakes and press residues	Residual proteins, unsaturated fatty acids, tocopherols, phytosterols, lignans or glucosinolates depending on source	Protein replacement, fatty acid modulation, antioxidant lipid protection	Milk, meat, eggs, fish fillets	Anti-nutritional factors; lipid oxidation; species-specific digestibility	[3,7,9]
Apple pomace and other fruit pomaces	Pectin, soluble fibre, phenolic acids, flavonoids, residual sugars	Gut fermentation, short-chain fatty acid production, antioxidant modulation	Monogastric products, ruminant products	Moisture; rapid spoilage; variable phenolic accessibility	[3,4]
Vegetable residues and mixed agro-industrial streams	Fibre, carotenoids, phenolics, minerals, residual proteins, organic acids	Local circular feeding, antioxidant modulation, fibre fermentation, partial feed replacement	Ruminant meat and milk, pork, poultry	High variability; safety controls; difficult attribution of effects in mixed matrices	[7,33,34]

**Table 2:** Representative evidence sources linking agro-industrial by-product feeding with animal-derived food quality or functional traits.

Species/Category	By-Product or Bioactive Source	Inclusion Level or Dose	Duration/Design	Product Evaluated	Main Product-Level or Biological Effects	Interpretation Strength	Reference
Dairy sheep	Spray-dried phenolic fraction from olive mill wastewater	25 g/kg concentrate	36 Sarda ewes; feeding trial for cheese production	Milk and cheese	Hydroxytyrosol, tyrosol and sulphate metabolites detected at higher levels in cheese from supplemented ewes	Strong: targeted metabolite detection in dairy product	[21]
Italian dairy products	No controlled feeding trial; survey of commercial cheeses	—	36 cheeses from ewe, goat and cow milk	Cheese	Hydroxytyrosol, tyrosol and sulphate metabolites detected; ewe cheeses richer in total detected phenolics than cow cheeses	Supportive: confirms analytical detectability of phenolic metabolites in cheese	[22]
Dairy cows	Pelleted citrus pulp	9% and 18% of diet DM	4 lactating Holstein cows; 4 × 4 Latin square	Milk	Increased total polyphenols, flavonoids and FRAP in milk; effects more evident at 9–18% DM	Moderate/strong: product-level antioxidant traits measured, but individual metabolites not fully traced	[23]
Dairy goats	Olive leaves	10% dietary supplementation	30 Saanen goats; feeding trial	Raw goat milk	Increased antioxidant potential and phenolic-related traits; reduction of lipolytic events and changes in volatile/lipolytic profile	Moderate: product-level traits measured, mechanism partly inferred	[48]
Dairy goats	Dried grape pomace	Up to 6% of diet DM	Dairy goat feeding trial	Milk	No depression of productive performance; limited changes in milk composition; potential modulation of milk fatty acid profile	Moderate: product-level FA response, limited mechanistic data	[49]
Dairy goats	Ensiled white grape pomace	0%, 5%, 10% and 15% of diet DM	88 Murciano-Granadina goats; isoenergetic and isoproteic diets	Milk	Evaluation of nutritional and technological milk traits under increasing grape pomace inclusion	Moderate: useful dose-response design; final effects to be interpreted by product endpoints	[50]
Lambs	Grape pomace	10% of diet DM	30 lambs; 45 days	Longissimus dorsi meat	Increased vaccenic and rumenic acids; lower hexanal and TBARS after cooked meat storage; improved oxidative stability	Strong: dose, product FA, VOCs and oxidation measured	[51]
Lambs	Silages from carrot, sweet potato, potato and tomato pomace mixtures	Replacement of 50% of concentrate DM	Lamb feeding trial	Carcass and meat	No impairment of growth performance, carcass traits, meat quality or in vitro methane emissions; lower feeding cost per kg live-weight gain	Moderate: strong circular-feeding relevance; limited molecular mechanism	[33]
Lambs	Dried tomato pomace	Used to reduce concentrate intake	Lamb feeding trial	Meat	Maintained growth and meat-quality traits while partially replacing concentrate; useful as circular feed ingredient	Moderate: product quality assessed, but carotenoid transfer not central	[52]
Laying hens	Tomato powder	5 and 10 g/kg diet	90 hens, 49 weeks old; 3 dietary groups	Eggs	Increased yolk carotenoids, vitamin A and yolk colour; reduced yolk MDA/lipid peroxidation	Strong: clear dose and direct yolk enrichment	[25]
Laying hens	Tomato waste	Variable inclusion levels across studies	Meta-analysis of laying hen trials	Eggs	Improved egg-quality traits and supported tomato waste as a useful natural carotenoid source	Supportive: synthesis of egg-quality evidence	[41]
Weaned/finishing pigs	Innovative silage from olive mill waste, grape pomace and feta cheese waste solids	0%, 5% and 10% of diet	Pig feeding trial	Meat and gut microflora	No negative effects on performance; modulation of gut microflora; positive effects on meat oxidative stability and fatty acid profile reported	Moderate: connects circular silage, microbiota and meat quality	[34]
Pigs	Silage from olive, winery and cheese by-products	Mixed silage, including olive mill waste and grape pomace	Pilot feeding study	Gut microbiota; indirect relevance for meat	Modulated gastrointestinal microbiota without major performance impairment	Preliminary: microbiota focus, product-level evidence limited	[53]
European sea bass	Red wine grape pomace	0.4% of diet	Juvenile sea bass; 5 weeks	Fillet	Improved oxidative-related responses and helped prevent oxidation during fillet storage; effects on physiological and immunological status	Moderate/strong: product oxidation and biological status measured	[54]
Common carp	Grape pomace	0%, 5% and 10% of diet	180 carp; 8 weeks	Fish body/biochemical composition	Most growth parameters unaffected; biochemical and physiological responses evaluated under 5–10% GP inclusion	Moderate: useful aquaculture dose-response, product-quality depth limited	[55]
Dairy cows/ruminants	Grape, pomegranate, olive and tomato by-products	5–40% of diet DM across studies	Review of dairy ruminant trials	Milk	Milk yield generally not depressed; positive effects on milk fatty acid profile reported across several by-products	Supportive: synthesis, not single in vivo trial	[6]
In vitro ruminant model	Dried grape pomace processed by micronization and air classification	Physical fractions obtained at 85/170 Hz and 200/240 psi	In vitro characterization	Feed matrix and rumen-oriented endpoints	Processing modified nutritional composition, phenolic content, in vitro digestibility and gas production	Supportive/mechanistic: matrix characterization before in vivo validation	[38]
Beef steers	Artichoke bracts silage	Finishing diet inclusion	Finishing beef trial with dry-aging evaluation	Dry-aged beef	Evaluated effects of a local vegetable by-product silage on dry-aged meat quality	Moderate/strong: in vivo product-level validation under post-mortem aging	[56]

**Table 3:** Main microbiome-mediated and host molecular pathways linking agro-industrial by-products with functional traits of animal-derived foods.

**By-Product Class**	**Main Bioactive or Matrix Feature**	**Main Microbial or Host Pathway**	**Suggested Analytical Markers**	**Expected Product-Level Effect**	**Key References**
Grape pomace and grape seeds	Condensed tannins, flavanols, anthocyanins, phenolic acids, seed lipids	Rumen protein binding; modulation of lipid biohydrogenation; antioxidant and inflammatory signalling	Rumen VFA, ammonia-N, biohydrogenation intermediates, vaccenic acid, rumenic acid, SOD, GPx, CAT, MDA, TBARS, VOCs	Improved milk/meat FA profile; lower lipid oxidation; modified volatile profile	[13,14,19,51]
Olive by-products	Hydroxytyrosol, tyrosol, oleuropein derivatives, secoiridoids, oleic acid	Microbial and host transformation of phenolics; phase-II conjugation; antioxidant modulation	Hydroxytyrosol, tyrosol, sulphate/glucuronide metabolites, FRAP, ORAC, MDA, milk/cheese phenolic profile	Phenolic metabolites in dairy products; improved antioxidant potential; possible lipid-stability effects	[20,21,22]
Citrus pulp and peel	Pectin, soluble carbohydrates, hesperidin, naringin, limonene	Rumen and gut fermentation; flavanone metabolism; SCFA production; possible volatile transfer	Acetate, propionate, butyrate, total polyphenols, flavonoids, FRAP, flavanone metabolites, limonene-related VOCs	Increased antioxidant-related milk traits; possible effects on fermentation and aroma	[4,23]
Tomato pomace	Lycopene, β-carotene, tocopherols, seed oil, fibre	Lipid-mediated absorption; carotenoid deposition; antioxidant protection in lipid-rich matrices	Lycopene, β-carotene, retinol/vitamin A, yolk color, yolk MDA, tocopherols, lipid oxidation	Yolk pigmentation; carotenoid enrichment; lower lipid peroxidation	[24,25,26,42]
Pomegranate and ellagitannin-rich residues	Ellagitannins, ellagic acid, anthocyanins	Microbial conversion to urolithins; antioxidant and anti-inflammatory signalling	Ellagic acid, urolithin A/B, phenolic acids, Nrf2-related markers, NF-κB-related markers, MDA	Potential antioxidant modulation; possible metabolite-mediated functionality	[9,11,27]
Cereal brans	Arabinoxylans, ferulic acid, p-coumaric acid, resistant fibre	Microbial fermentation of fibre; enzymatic release of bound phenolic acids; SCFA production	Ferulic acid, p-coumaric acid, arabinoxylans, acetate, propionate, butyrate, gut microbiota, antioxidant capacity	Indirect modulation through gut/rumen fermentation; possible antioxidant effects	[28,29,30]
Brewer’s spent grain	Arabinoxylans, cellulose, lignin, residual proteins, bound phenolic acids	Fibre fermentation; phenolic release after fermentation/enzymatic treatment; microbial protein contribution	NDF, ADF, arabinoxylans, ferulic acid, p-coumaric acid, microbial metabolites, digestibility, SCFA	Nutrient recovery; possible gut or rumen modulation; product effects still underexplored	[31,32,104]
Oilseed cakes and press residues	Residual proteins, unsaturated fatty acids, tocopherols, phytosterols	Lipid supply; host fatty acid remodeling; oxidative balance; protein replacement	FA profile, PUFA, MUFA, tocopherols, peroxide value, TBARS, SCD, FASN, PPAR-related markers	Improved FA profile of milk, meat or eggs; risk of oxidation if antioxidant protection is low	[6,9,16]
Mixed ensiled by-products	Organic acids, residual phenolics, fibre, proteins, microbial metabolites	Controlled fermentation; gut microbiota modulation; SCFA production; possible antioxidant effects	Lactic acid, acetic acid, pH, microbial counts, gut microbiota, SCFA, meat TBARS, FA profile	Circular feed use; gut modulation; possible meat-quality effects	[33,34,53]
Aquafeed by-product strategies	Phenolics, carotenoids, alternative lipids, fibre fractions	Gut microbiota modulation; antioxidant defense; lipid remodeling in fillet	Fillet FA profile, MDA/TBARS, antioxidant enzymes, gut microbiota, fillet VOCs, sensory traits	Improved oxidative stability; possible modulation of fillet lipid quality	[54,55,85]
Physically fractionated grape pomace	Fibre, phenolics, seed lipids, particle-size-dependent fractions	Matrix accessibility; in vitro ruminal digestion; fermentation kinetics	Phenolic profile, NDF/ADF, in vitro digestibility, gas production	Improved tailoring of winery residues before animal validation	[38]
Artichoke bracts and Mediterranean vegetable residues	Fibre, phenolics, ensilable biomass	Fibre fermentation, antioxidant modulation, replacement of conventional roughage	NDF, ADF, phenolics, meat pH, color, WHC, TBARS, FA profile, aging traits	Circular roughage source; possible modulation of dry-aged meat quality	[56]

## Data Availability

No new data were created or analyzed in this study. Data sharing is not applicable to this article.

## Supplementary Materials

The following supporting information can be downloaded at: https://www.mdpi.com/article/10.3390/molecules31152710/s1: Table S1: Full search strategies used in Scopus, PubMed/MEDLINE, and Web of Science Core Collection; Table S2: Semi-quantitative evidence-strength framework for interpreting by-product-mediated functional animal-derived foods; Table S3: Comparative digestive, microbial and host-mediated transformation pathways of by-product-derived bioactives in ruminant and monogastric food-producing animals; Table S4: Standardized data extraction template used for data charting and evidence synthesis; Table S5: Processing-route comparison and life-cycle-related indicators for circular valorization of agro-industrial by-products in animal feeding.
