# Supplementary material for: Transformation of Agro-Industrial By-Products into High-Value Animal-Derived Foods: Bioactive Compounds, Microbiome-Mediated Biotransformation, Metabolomic Traceability and Circular Valorization
Source: Molecules. 2026 Aug 4;31(15):2710. doi: 10.3390/molecules31152710 (PMC13467954; doi:10.3390/molecules31152710)
Supplement: Supplementary file 1 [file molecules-31-02710-s001.zip › molecules-4452583-supplementary.pdf]

Table S1. Full search strategies used in Scopus, PubMed/MEDLINE, and Web of Science Core Collection.

Database	Search field and filters	Search string
Scopus	TITLE-ABS-KEY; publication years 2011–2026; English language	TITLE-ABS-KEY ((“agro-industrial by-product*” OR “agri-food by-product*” OR “food processing residue*” OR “food-chain residue*” OR “agro-industrial residue*” OR “agri-food residue*” OR “grape pomace” OR “olive pomace” OR “olive cake” OR “olive mill wastewater” OR “olive leaves” OR “tomato pomace” OR “tomato waste” OR “citrus pulp” OR “citrus peel*” OR “cereal bran” OR “wheat bran” OR “brewer* spent grain” OR “oilseed cake*” OR “vegetable residue*” OR “fruit pomace”) AND (“animal feed” OR feed* OR livestock OR ruminant* OR “dairy cow*” OR cattle OR sheep OR goat* OR lamb* OR pig* OR swine OR poultry OR chicken* OR “laying hen*” OR rabbit* OR fish OR aquaculture) AND (polyphenol* OR phenolic* OR tannin* OR flavonoid* OR carotenoid* OR tocopherol* OR terpene* OR “fatty acid*” OR peptide* OR metabolomic* OR lipidomic* OR microbiome OR microbiota OR fermentation OR biohydrogenation OR “short-chain fatty acid*” OR SCFA OR urolithin* OR equol OR “phenolic metabolite*”) AND (“milk quality” OR cheese OR “meat quality” OR “egg quality” OR “fish quality” OR fillet* OR “animal-derived food*” OR “food of animal origin” OR “oxidative stability” OR “fatty acid profile” OR “colour stability” OR “color stability” OR “volatile compound*” OR “sensory quality” OR shelf-life OR “functional food*”)) AND PUBYEAR > 2010 AND PUBYEAR < 2027 AND LIMIT-TO (LANGUAGE, “English”)
PubMed/MEDLINE	Title/Abstract; publication dates 01 January 2011–15 June 2026; English language	((“agro-industrial by-product*” OR “agri-food by-product*” OR “food processing residue*” OR “food-chain residue*” OR “agro-industrial residue*” OR “agri-food residue*” OR “grape pomace” OR “olive pomace” OR “olive cake” OR “olive mill wastewater” OR “olive leaves” OR “tomato pomace” OR “tomato waste” OR “citrus pulp” OR “citrus peel” OR “citrus peels” OR “cereal bran” OR “wheat bran” OR “brewer spent grain” OR “brewers spent grain” OR “brewer’s spent grain” OR “oilseed cake” OR “oilseed cakes” OR “vegetable residue” OR “vegetable residues” OR “fruit pomace”) AND (“animal feed” OR feed* OR livestock OR ruminant* OR “dairy cow” OR “dairy cows” OR cattle OR sheep OR goat* OR lamb* OR pig* OR swine OR poultry OR chicken* OR “laying hen” OR “laying hens” OR rabbit* OR fish OR aquaculture) AND (polyphenol* OR phenolic* OR tannin* OR flavonoid* OR carotenoid* OR tocopherol* OR terpene* OR “fatty acid” OR “fatty acids” OR peptide* OR metabolomic* OR lipidomic*

Database	Search field and filters	Search string
		OR microbiome OR microbiota OR fermentation OR biohydrogenation OR “short-chain fatty acid” OR “short-chain fatty acids” OR SCFA OR urolithin* OR equol OR “phenolic metabolite” OR “phenolic metabolites”) AND (“milk quality” OR cheese OR “meat quality” OR “egg quality” OR “fish quality” OR fillet* OR “animal-derived food” OR “animal-derived foods” OR “food of animal origin” OR “foods of animal origin” OR “oxidative stability” OR “fatty acid profile” OR “colour stability” OR “color stability” OR “volatile compound” OR “volatile compounds” OR “sensory quality” OR shelf-life OR “functional food” OR “functional foods”)) AND (“2011/01/01” : “2026/06/15”) AND English
Web of Science Core Collection	Topic field; publication years 2011–2026; English language	TS=((“agro-industrial by-product*” OR “agri-food by-product*” OR “food processing residue*” OR “food-chain residue*” OR “agro-industrial residue*” OR “agri-food residue*” OR “grape pomace” OR “olive pomace” OR “olive cake” OR “olive mill wastewater” OR “olive leaves” OR “tomato pomace” OR “tomato waste” OR “citrus pulp” OR “citrus peel*” OR “cereal bran” OR “wheat bran” OR “brewer* spent grain” OR “oilseed cake*” OR “vegetable residue*” OR “fruit pomace”) AND (“animal feed” OR feed* OR livestock OR ruminant* OR “dairy cow*” OR cattle OR sheep OR goat* OR lamb* OR pig* OR swine OR poultry OR chicken* OR “laying hen*” OR rabbit* OR fish OR aquaculture) AND (polyphenol* OR phenolic* OR tannin* OR flavonoid* OR carotenoid* OR tocopherol* OR terpene* OR “fatty acid*” OR peptide* OR metabolomic* OR lipidomic* OR microbiome OR microbiota OR fermentation OR biohydrogenation OR “short-chain fatty acid*” OR SCFA OR urolithin* OR equol OR “phenolic metabolite*”) AND (“milk quality” OR cheese OR “meat quality” OR “egg quality” OR “fish quality” OR fillet* OR “animal-derived food*” OR “food of animal origin” OR “oxidative stability” OR “fatty acid profile” OR “colour stability” OR “color stability” OR “volatile compound*” OR “sensory quality” OR shelf-life OR “functional food*”)) AND PY=(2011-2026) AND LA=(English)
Scopus, PubMed/MEDLINE, Web of Science Core Collection and reference checking	Targeted check within eligible full texts, reference lists and contextual safety	(“agro-industrial by-product*” OR “agri-food by-product*” OR “food processing residue*” OR “fruit pomace” OR “vegetable residue*” OR “cereal bran” OR “oilseed cake*” OR “olive pomace” OR “grape pomace” OR “tomato pomace” OR “citrus pulp”) AND (“feed safety” OR contaminant* OR mycotoxin*

Database	Search field and filters	Search string
	sources; English language; 2011–2026 when applicable	OR “pesticide residue*” OR “heavy metal*” OR dioxin* OR PCB* OR “microbial contamination” OR spoilage OR “biogenic amine*” OR “oxidized lipid*” OR “solvent residue*” OR “anti-nutritional factor*” OR carry-over

Table S2. Semi-quantitative evidence-strength framework for interpreting by-product-mediated functional animal-derived foods.

Table S2A. Scoring domains

Domain	Score 0	Score 1	Score 2
**1. By-product characterization and active-dose definition**	By-product described only by name or broad category; no information on composition, processing or inclusion on a dry-matter basis.	Partial characterization available, such as proximate composition, inclusion level or processing method, but limited information on bioactive fractions or active dose.	Adequate characterization of the final ingredient as administered, including origin, processing/stabilization method, inclusion level on a dry-matter basis and relevant bioactive fractions or active-dose indicators.
**2. Feeding design and biological relevance**	No in vivo feeding trial, or only chemical/extraction/in vitro data not linked to animal feeding.	In vivo feeding trial available, but with limited control of basal diet, inclusion level, feeding duration or animal response.	Realistic in vivo feeding trial with adequate control diet, biologically relevant inclusion level, feeding duration and assessment of animal performance or physiological response.
**3. Final animal-derived product analysis**	No analysis of milk, cheese, meat, eggs, fish or derived products.	Basic product analysis only, such as proximate composition, yield, general quality traits or broad antioxidant capacity.	Direct product-level analysis of relevant traits, such as fatty acid profile, oxidation markers, colour stability, volatile compounds, sensory traits, shelf-life, specific metabolites or compound enrichment.
**4. Microbial, metabolic or host-mediated mechanistic evidence**	No microbiome, metabolite, host-response or mechanistic endpoint.	Limited mechanistic evidence, such as rumen/gut fermentation variables, general oxidative markers, selected blood metabolites or broad microbiota descriptors.	Mechanistic evidence linked to the proposed pathway, including microbial composition or function, metabolomics/lipidomics, biohydrogenation intermediates, host metabolic markers or targeted metabolite tracing.
**5. Safety and circularity reporting**	No information on feed safety, contaminants, hygiene, processing feasibility or circular value.	Partial information on safety or circularity, such as feed substitution, local availability, processing method or general sustainability considerations.	Safety and circularity explicitly considered, including feed hygiene, spoilage risk, mycotoxins, pesticide residues, heavy metals, solvent residues, substitution of conventional feedstuffs, processing intensity, scalability or life-cycle-related indicators.

Table S2B. Total score and evidence level

Total score	Evidence level	Interpretation
**8–10**	Strong evidence	The study provides integrated evidence connecting by-product characterization, realistic feeding, mechanistic intermediates and final product-level outcomes. Functional enrichment, metabolite-mediated transfer or indirect quality modulation can be discussed with confidence, while acknowledging the need for validation across farms, breeds, diets and production systems.
**6–7**	Moderate evidence	The study demonstrates relevant product-level effects in vivo, but the mechanism is only partly clarified or some information on active dose, processing, safety or circularity is incomplete. Functional interpretation is possible but should remain cautious.
**3–5**	Weak evidence	The study suggests a possible effect on product quality, but evidence is incomplete because of limited by-product characterization, unclear active dose, weak mechanistic endpoints or incomplete product analysis. These studies are useful for hypothesis generation but are insufficient for strong functional-food claims.
**0–2**	Insufficient evidence	The study reports only chemical composition, extraction yield, in vitro antioxidant capacity, simulated digestion, general digestibility or animal performance without measurable connection to the final animal-derived product. These data support ingredient potential, but not functional animal-derived food production.

Table S2C. Claim type and suitable wording

Claim type	Minimum evidence required	Suitable wording
**Direct enrichment**	A defined native compound or closely related molecule is quantified and increased in the edible product using appropriate analytical methods.	"Dietary supplementation increased specific bioactive compounds in the final product."
**Metabolite-mediated transfer**	A microbial or host-derived metabolite linked to the by-product is detected and quantified in the edible product.	"The by-product supported the appearance or increase of specific metabolites in the final product."
**Indirect quality modulation**	Product quality improves without clear detection of a transferred compound, through changes in lipid metabolism, oxidative stability, microbial fermentation or host response.	"The by-product modulated product quality, probably through indirect microbial or host-mediated mechanisms."
**Ingredient potential only**	The by-product contains potentially bioactive molecules or improves in vitro indicators, but final product data are absent.	"The by-product shows potential as a bioactive feed resource, but its effect on animal-derived foods remains unproven."

Table S3. Comparative digestive, microbial and host-mediated transformation pathways of by-product-derived bioactives in ruminant and monogastric food-producing animals.

Compound or pathway	Ruminants	Monogastric species	Main mechanistic difference
**General digestive and microbial context**			
Principal site of microbial transformation	Rumen.	Distal intestine, particularly the ceca and colon, with differences among pigs, poultry, rabbits and fish.	In ruminants, microbial transformation occurs mainly before small-intestinal absorption. In monogastric species, partial digestion and absorption can occur in the upper gastrointestinal tract, whereas poorly absorbed compounds are transformed mainly in the distal intestine.
Matrix disruption and release of polyphenols and related phenolic fractions	Microbial degradation in the rumen extensively releases fibre-bound phenolic fractions before intestinal absorption.	Partial release may occur during enzymatic digestion in the upper gastrointestinal tract, whereas remaining fibre-bound fractions reach the hindgut.	In ruminants, release is mainly ruminal and pre-absorptive; in monogastric species, release is divided between upper-gut digestion and hindgut microbial activity.
Flavonoids			
Flavonoid transformation	Flavonoids are transformed predominantly in the rumen through microbial deglycosylation, de-esterification, reduction, dehydroxylation, decarboxylation and aromatic ring cleavage. Rumen microorganisms extensively degrade flavonoids before intestinal absorption, producing aglycones and smaller phenolic metabolites.	Flavonoids are transformed mainly in the hindgut through deglycosylation, de-esterification, demethylation, dehydroxylation, decarboxylation and ring cleavage. Some flavonoids or aglycones may be absorbed in the small intestine, whereas unabsorbed compounds undergo further microbial transformation in the hindgut.	Similar reaction classes may occur in both digestive systems, but at different anatomical sites and before or after potential small-intestinal absorption. In ruminants, flavonoids undergo extensive microbial transformation in the rumen before absorption; in monogastric species, microbial transformation in the hindgut follows partial upper-gut digestion and absorption.
Isoflavone transformation	Specific rumen microorganisms may convert daidzein into equol, which can subsequently enter blood circulation and be transferred to milk.	Equol is produced by specific intestinal bacteria, mainly in the hindgut, and production varies among species and individuals.	In ruminants, equol formation occurs mainly in the rumen. In monogastric species, equol formation occurs mainly in the hindgut and depends on the presence of equol-producing microbiota. Production depends on precursor availability and specific microbial functions, not only on dietary concentration.
**Phenolic acids**			

Compound or pathway	Ruminants	Monogastric species	Main mechanistic difference
Native and fibre-bound phenolic acids	Phenolic acids are released from plant cell-wall structures by rumen microbial esterases, followed by further microbial transformation before absorption.	Free phenolic acids may be absorbed in the small intestine, whereas fibre-bound forms reach the hindgut and are released by microbial enzymes.	In ruminants, release is mainly pre-absorptive and occurs earlier. In monogastric species, phenolic acid availability results from combined small-intestinal absorption and hindgut microbial release.
Microbial phenolic metabolites	Ruminal degradation of tannins and flavonoids produces low-molecular-weight aromatic acids before intestinal absorption.	Unabsorbed polyphenols are transformed mainly in the hindgut into phenylacetic, phenylpropionic, hydroxybenzoic and hydroxycinnamic derivatives.	Similar metabolites may be formed in both digestive systems, but their principal site of formation differs: predominantly before absorption in ruminants and after partial upper-gut digestion or absorption in monogastric species.
Tannins			
Hydrolysable tannins and ellagitannins	Hydrolysable tannins may be hydrolysed by microbial tannases to gallic or ellagic acid; ellagic acid may subsequently be converted to urolithins by specific gastrointestinal microorganisms.	Unabsorbed ellagitannins and ellagic acid may be converted mainly by hindgut microbiota into urolithins.	In ruminants, transformation may begin before intestinal absorption. In monogastric species, urolithin formation occurs mainly in the hindgut. Urolithin production is microbiome- and metabotype-dependent.
Condensed tannins	Condensed tannins interact strongly with proteins and microbial cells; they may affect fibre degradation, methane formation and lipid biohydrogenation, although microbial degradation may be incomplete.	Condensed tannins are poorly absorbed in the upper gastrointestinal tract, and their hindgut transformation depends on tannin structure and animal species.	In ruminants, pre-absorptive effects on rumen fermentation dominate. In monogastric species, hindgut effects are more relevant, although effects on upper-gut digestibility may also occur.
Tannin–protein interactions	Tannins can form complexes with dietary proteins in the rumen, reducing proteolysis and ammonia formation and increasing the flow of rumen-undegraded protein to the small intestine. Excessive levels may reduce protein and fibre digestibility and feed intake.	Tannins can bind dietary proteins and digestive enzymes in the stomach and small intestine, generally reducing protein digestion and absorption. Unabsorbed tannins and some tannin–protein complexes may reach the hindgut and affect microbial activity.	In ruminants, moderate tannin–protein binding may protect protein from ruminal degradation and increase intestinal protein flow. In monogastric species, tannin–protein binding more often reduces protein digestion and absorption.
**Polyphenol- and tannin-related end-products**			

Compound or pathway	Ruminants	Monogastric species	Main mechanistic difference
Host transformation after absorption	Microbial metabolites absorbed from the gastrointestinal tract undergo phase-II conjugation, particularly hepatic and tissue glucuronidation, sulphation and methylation, before secretion into milk or deposition in tissues.	Native compounds absorbed in the upper gut and microbial metabolites absorbed from the hindgut undergo similar host phase-II conjugation, followed by tissue or egg deposition, urinary excretion or biliary excretion.	In both ruminant and monogastric species, animal-derived foods generally contain conjugated or microbial–host co-metabolites rather than native feed compounds.
Representative microbial and microbial–host metabolites	Phenolic acids; equol; urolithins; hydroxytyrosol/tyrosol-related metabolites; other aromatic products.	Phenylacetic, phenylpropionic, hydroxybenzoic and hydroxycinnamic derivatives; urolithins; equol; other microbial–host co-metabolites.	The classes of metabolites partly overlap, but their principal site of formation and relative contribution differ between digestive systems.
Fibre fermentation and major end-products	Extensive ruminal fermentation produces acetate, propionate and butyrate and influences microbial protein synthesis, ammonia formation, hydrogen utilization and methane production.	Hindgut or cecal fermentation produces mainly acetate, propionate and butyrate, with proportions depending on substrate and species.	In ruminants, fermentation products are major contributors to energy supply and milk-fat synthesis. In monogastric species, these pathways primarily affect gut barrier function, immune tone and systemic metabolism.
Predominant product-level pathway	Effects on milk and meat are mediated mainly through ruminal fermentation, nitrogen metabolism, lipid biohydrogenation and metabolite transfer to milk, cheese or meat.	Effects on meat, eggs or fish products more often combine direct absorption and deposition with hindgut-derived metabolites and systemic modulation of oxidative, inflammatory and lipid-metabolic pathways.	Ruminant products are particularly sensitive to microbial modification before absorption. Monogastric products may more directly reflect dietary supply, especially for lipophilic compounds.
**Interactions with other microbial metabolic pathways**			
Polyphenols, tannins and unsaturated lipid transformation	Tannins and flavonoids may modify ruminal microbial lipolysis and biohydrogenation, influencing the formation of vaccenic and rumenic acid intermediates.	The effect of polyphenols on lipid transformation is less direct and less consistently demonstrated than in ruminants. Dietary fatty acids are more directly absorbed and subsequently modified by host metabolism.	In ruminants, these processes can strongly affect milk and meat fatty acid profiles. In monogastric species, product fatty acid profiles more directly reflect dietary lipid supply and host lipid metabolism.

Compound or pathway	Ruminants	Monogastric species	Main mechanistic difference
Polyphenols, tannins and methane/hydrogen metabolism	Polyphenols, particularly tannins, may affect methanogens, protozoa-associated methanogenesis and hydrogen distribution in the rumen.	This interaction has lower product-level relevance than in ruminants, because hindgut methane formation is generally less important for feed efficiency and animal-derived food quality.	In ruminants, tannin-rich polyphenol fractions can moderately redirect hydrogen utilization and reduce methane formation.
**Lipophilic bioactives with limited or indirect microbial biotransformation**			
Carotenoids	Carotenoids are not primarily transformed by rumen microbiota. Their biological relevance depends mainly on matrix release, protection from oxidation, lipid-mediated intestinal absorption and subsequent deposition in milk fat or tissues. Ruminal digestion and diet composition may indirectly affect carotenoid availability.	Carotenoids may be released during digestion, incorporated into lipid micelles, absorbed in the small intestine and deposited particularly efficiently in egg yolk or lipid-rich tissues. In laying hens, this pathway is one of the clearest examples of direct feed-to-food enrichment.	In ruminants, carotenoid transfer is generally less direct and may be influenced by rumen conditions, lipid supply and tissue metabolism. In monogastric species, carotenoids can be more directly absorbed and deposited, leading to measurable yolk pigmentation and enrichment.
Tocopherols and other lipophilic antioxidants	Tocopherols may contribute to antioxidant protection of milk and meat lipids, but their effects depend on dietary lipid supply, oxidative status and tissue deposition, and are not primarily dependent on microbial transformation.	Tocopherols may be absorbed with dietary lipids and contribute to antioxidant protection in eggs, meat or fish tissues, especially when diets increase the supply of unsaturated fatty acids.	In both digestive systems, tocopherols act mainly through absorption, deposition and antioxidant protection rather than microbial biotransformation.
Terpenes, terpenoids and volatile compounds	Citrus- and olive-derived terpenes may be partly modified in the rumen, affect palatability and ruminal microbial activity, and contribute indirectly to milk or meat aroma through changes in fermentation and volatile formation.	Volatile terpenes may be absorbed, metabolized or excreted, with potential effects on palatability, gut microbiota and aroma-related traits of eggs, meat or fish, depending on dose and matrix.	These compounds are less directly connected with microbial biotransformation than polyphenols, but they are relevant for aroma, palatability and sensory quality. Their effects are dose- and matrix-dependent and should be interpreted together with volatile-profile and sensory data.

Compound or pathway	Ruminants	Monogastric species	Main mechanistic difference
Unsaturated fatty acids and phytosterols	Dietary unsaturated fatty acids from seed-rich residues are extensively modified by ruminal lipolysis and biohydrogenation before absorption, producing intermediates such as vaccenic and rumenic acids that may influence milk and meat fatty acid profiles. Phytosterols and minor lipid compounds may contribute to lipid metabolism and oxidative balance, but their transfer is less consistently evaluated.	Dietary fatty acids from by-products are more directly absorbed and incorporated into tissues, egg yolk or fish tissues, although host metabolism can further remodel lipid profiles. Phytosterols and minor lipid compounds may contribute to lipid quality and antioxidant-related responses.	In ruminants, product fatty acid composition is strongly shaped by ruminal biohydrogenation. In monogastric species, product lipid profiles more directly reflect dietary lipid supply and host lipid metabolism.
**Other compounds**			
Residual proteins and peptides	Residual proteins from oilseed cakes, brewer's spent grain, legume fractions or seed fractions may be degraded by rumen microorganisms to peptides, amino acids and ammonia, contributing to microbial protein synthesis.	Residual proteins and peptides are mainly digested enzymatically in the stomach and small intestine. High-fibre matrices may reduce digestibility unless processing, fermentation or enzymatic treatment improves protein accessibility.	In ruminants, protein fractions are strongly integrated with rumen nitrogen metabolism and microbial protein synthesis. In monogastric species, their value depends more directly on digestibility, amino acid availability and processing-related improvement of matrix accessibility.

Table S4. Standardized data extraction template used for data charting and evidence synthesis.

Extraction domain	Variable extracted	Description or examples
Bibliographic information	Author(s), year, journal, country	Basic information used to identify the study and its geographical or production context.
Study type	Original in vivo trial, in vitro study, simulated digestion, fermentation study, extraction study, review, meta-analysis or contextual source	Used to distinguish direct product-level evidence from mechanistic or background evidence.
Animal species and category	Dairy cows, sheep, goats, beef cattle, pigs, poultry, laying hens, rabbits, fish or other aquaculture species	Used to classify evidence according to digestive physiology and final animal-derived product.
Production system	Dairy, meat, egg, aquaculture or mixed production system	Used to connect feeding strategy with the final product evaluated.
By-product type	Grape pomace, olive pomace, olive leaves, olive mill wastewater, tomato pomace, citrus pulp, cereal bran, brewer's spent grain, oilseed cake, vegetable residues or other by-products	Used to classify by-products according to industrial origin and dominant functional fractions.
Botanical or industrial origin	Plant species, cultivar when available, industrial process and recovered fraction	Used to evaluate matrix variability and reproducibility across studies.
Processing or stabilization method	Fresh use, drying, milling, micronization, air classification, ensiling, co-ensiling, fermentation, enzymatic treatment, extraction or encapsulation	Used to evaluate how processing may preserve, release, concentrate or degrade bioactive fractions.
Storage and handling conditions	Moisture level, storage time, storage temperature, packaging or preservation strategy when reported	Used to interpret stability, spoilage risk and preservation of bioactive compounds.
Dietary inclusion level	Inclusion level as-fed and/or on a dry-matter basis	Used to compare feeding strategies and define biological relevance.
Active-dose indicators	Polyphenol dose, tannin dose, carotenoid dose, lipid fraction, antioxidant capacity or other bioactive markers	Used to avoid interpreting by-products only by name or broad category.
Basal diet characteristics	Forage-to-concentrate ratio, lipid level, protein level, energy density, fibre fractions or main ingredients	Used to evaluate whether the response depends on the whole diet rather than only the by-product.
Feeding duration	Duration of supplementation or experimental period	Used to assess whether the exposure period was sufficient to affect animal metabolism or product quality.
By-product characterization	Proximate composition, fibre fractions, lipid profile, polyphenol profile, carotenoids, tocopherols, antioxidant capacity, anti-nutritional factors or contaminants	Used to assess the quality of matrix characterization.

Extraction domain	Variable extracted	Description or examples
Microbiome-related endpoints	Ruminal or intestinal microbiota, fermentation variables, biohydrogenation indicators, short-chain fatty acids, microbial metabolites or omics endpoints	Used to evaluate microbial transformation and mechanistic support.
Host-response endpoints	Productive performance, metabolic profile, oxidative-stress markers, inflammatory markers, immune response, transcriptomics, metabolomics, lipidomics or proteomics	Used to connect feed composition with animal physiology.
Product-quality endpoints	Milk composition, cheese traits, meat quality, egg quality, fish fillet quality, fatty acid profile, oxidation markers, antioxidant capacity, colour stability, volatile compounds, sensory traits, shelf-life or technological properties	Used to evaluate whether the by-product influenced the final animal-derived food.
Compound transfer or metabolite tracing	Detection of native dietary compounds, microbial metabolites or host-derived metabolites in milk, cheese, meat, eggs, fish or derived products	Used to distinguish direct enrichment or metabolite-mediated transfer from indirect quality modulation.
Safety endpoints	Feed hygiene, spoilage risk, mycotoxins, pesticide residues, heavy metals, microbial contamination, anti-nutritional factors or solvent residues	Used to evaluate feed safety and limitations for practical application.
Circularity and feasibility endpoints	Local availability, replacement of conventional feedstuffs, processing intensity, transport distance, scalability, cost, life-cycle-related indicators or waste-reduction potential	Used to evaluate circular valorization beyond simple ingredient substitution.
Evidence score	Score from 0 to 10 according to Supplementary Table S2A–C	Used to harmonize interpretation of heterogeneous evidence.
Main interpretation	Direct enrichment, metabolite-mediated transfer, indirect quality modulation, ingredient potential only or insufficient evidence	Used to classify the strength and type of functional-food interpretation supported by each source.
Main limitations	Missing matrix characterization, unclear active dose, absence of product-level endpoints, lack of microbiome/metabolomics data, limited safety information or weak circularity assessment	Used to identify recurrent knowledge gaps.

Table S5. Processing-route comparison and life-cycle-related indicators for circular valorization of agro-industrial by-products in animal feeding**.**

Processing route	Main objective	Life-cycle-related indicators to consider	Expected circular advantage	Main environmental or economic trade-offs	Most suitable use
**Local use of fresh by-products**	Rapid nutrient recovery with minimal processing	Distance between producer and farm; time from production to feeding; storage losses; hygiene control; replacement of conventional feedstuffs	Low processing energy; strong territorial integration; reduced waste-disposal pressure	High spoilage risk; seasonal availability; variable composition; limited transport radius	Nearby farms using high-moisture residues quickly and safely
**Local ensiling or co-ensiling**	Stabilization of moist residues for medium-term use	Dry matter adjustment; fermentation quality; additive use; storage losses; transport distance; substitution ratio; feed hygiene	Preserves local circularity while reducing spoilage; useful for high-moisture residues	Requires suitable co-substrates, storage infrastructure and fermentation control; poor ensiling can reduce palatability or safety	Fruit pomaces, tomato pomace, citrus pulp, vegetable residues and mixed local by-products, especially in ruminant systems
**Drying and milling**	Shelf-life extension, transportability and easier diet formulation	Energy demand for drying; moisture removal efficiency; storage stability; bioactive compound retention; transport distance; cost per unit of dry matter	Improves stability and allows inclusion in formulated feeds	Energy-intensive drying may reduce circular benefit; heat, oxygen and light may degrade carotenoids, lipids or polyphenols	By-products requiring stable supply, reduced moisture and standardized inclusion
**Fermentation or enzymatic upgrading**	Improved digestibility, release of bound compounds or reduction of anti-nutritional constraints	Microbial strain or enzyme system; process duration; energy and water use; stabilization after treatment; safety control; scalability	Can increase bioaccessibility and create a higher-value feed ingredient	Requires process standardization, quality control and post-treatment stabilization; laboratory benefits may not translate into farm-scale feasibility	Fibre-rich or polyphenol-bound matrices when improved accessibility is needed
**Fractionation or extraction**	Concentration of selected bioactive fractions	Solvent or water use; solvent recovery; extraction yield; purification needs; residue management; regulatory status; active-dose standardization	Allows targeted supplementation and mechanistic testing	May reduce nutrient-recovery value; can increase processing cost and environmental burden; contaminants may be concentrated if purification is inadequate	Standardized bioactive supplementation or studies requiring defined active compounds
**Long-distance transport of processed by-products**	Use of residues outside the production area	Transport distance; load density; moisture content; packaging; shelf-life; substituted ingredient; market value	Expands availability beyond local production zones	Transport emissions and costs may offset circular benefits, especially for bulky or moist materials	Dried, stable or high-value ingredients with clear nutritional or functional benefit

Processing route	Main objective	Life-cycle-related indicators to consider	Expected circular advantage	Main environmental or economic trade-offs	Most suitable use
**Use of purified or concentrated extracts**	Functional modulation through defined molecules	Active compound concentration; extraction intensity; solvent residues; regulatory classification; cost; evidence of product-level effect	High standardization and dose control	Lowest nutrient-recovery contribution; higher processing burden; greater need for safety and regulatory validation	Targeted experimental or commercial applications where direct product-quality effects are demonstrated
